# Evaluating the pentapharmacological potency of otamixaban against lung cancer CDK2, transferase, oxidoreductase and signalling proteins

**DOI:** 10.1371/journal.pone.0334013

**Published:** 2025-10-09

**Authors:** Hanadi M. Baeissa, Tahani Bakhsh, Afnan Mohammed Shakoori, Ghadir Sindi, Randa Mohammed Zaki, Mohammad Azhar Kamal, Hadeel A. Alsufyani, Alaa Hamed Habib, Arwa Ishaq A. Khayyat, Misbahuddin Rafeeq, Deeba Shamim Jairajpuri

**Affiliations:** 1 Department of Biological Sciences, College of Science, University of Jeddah, Jeddah, Saudi Arabia; 2 Department of Clinical Laboratory Sciences, Faculty of Applied Medical Sciences, Umm Al-Qura University, Makkah, Kingdom of Saudi Arabia; 3 Department of Pharmaceutics, College of Pharmacy, Prince Sattam Bin Abdulaziz University, Al-Kharj, Saudi Arabia; 4 Chair of Clinical Physiology Department, Medical College, King Abdulaziz University, Jeddah, Saudi Arabia; 5 Department of Physiology, Faculty of Medicine, King Abdulaziz University, Jeddah, Saudi Arabia; 6 Department of Biochemistry, College of Science, King Saud University, Riyadh, Saudi Arabia; 7 Department of Pharmacology, Basic Medical Sciences Division, College of Medicine, Dhofar University, Salalah, Oman; 8 Department of Medical Biochemistry, College of Medicine and Health Sciences, Arabian Gulf University, Manama, Kingdom of Bahrain; University of Nairobi Faculty of Health Sciences, KENYA

## Abstract

Lung cancer remains the leading cause of cancer-related mortality worldwide. With approximately 2.2 million new cases and 1.8 million deaths annually, it contributes to 18% of all cancer fatalities. The high mortality rate, limited accessibility to affordable treatments, and the emergence of drug resistance necessitated the development of novel therapeutic strategies. In this study, we employed an HTVS, SP and XP-based molecular docking and MM/GBSA calculations to identify a potential multitargeted drug candidate from the DrugBank library against five key (Penta) lung cancer-associated proteins, including CDK2 (1AQ1), Transferase(1JWH, 1K3A), Oxidoreductase (4XZL) and Signalling (2DVJ) enzymes that led to the identification of Otamixaban, exhibiting favourable docking and MM/GBSA scores ranging from −11.841 to −6.52, and −69.96 to −45.22 kcal/mol. Molecular Interaction Fingerprints analysis was performed to gain deeper insights into its binding interactions that reveal key residues with high interaction frequencies, including 12VAL, 8LEU, 7PHE, 6LYS, 5ASP, 5GLN, and 5GLU. Pharmacokinetic evaluations confirmed drug-likeness, and its ADMET properties were consistent with standard drug approval benchmarks, QM computations using DFT further reinforced the stability and reactivity of the identified compound. The thermodynamic role of water molecules in ligand binding was assessed through 5 ns WaterMap analysis, supporting the hypothesis that Otamixaban effectively interacts with the binding pockets of multiple target proteins. Further, a 100-ns MD simulation was conducted to ensure the stability and efficacy of the drug candidate under physiological conditions in an explicit TIP3P water environment. The results demonstrated minimal structural deviations and fluctuations, with strong intermolecular interactions persisting throughout the simulation. Further evaluation of 1000 simulation frames from trajectories provided comprehensive insights into the total complex energy and binding free energy via MM/GBSA calculations, reinforcing the potential of Otamixaban as a robust multitargeted drug candidate. Despite these promising computational findings, experimental validation through in vitro and in vivo studies is crucial to confirm its therapeutic efficacy and clinical viability.

## 1. Introduction

Lung cancer is one of the most prevalent and lethal malignancies worldwide, representing a significant global health burden. It continues to be the leading cause of cancer-related mortality globally, accounting for around 2.2 million new cases and 1.8 million deaths each year, which represents approximately 18% of all cancer-related fatalities [1,2]. It originates in the lungs and is characterised by uncontrolled cell proliferation, ultimately leading to metastasis and organ failure if left untreated [3]. The disease is broadly classified into two major types: non-small cell lung cancer (NSCLC) and small cell lung cancer (SCLC) [4]. NSCLC accounts for approximately 85% of all lung cancer cases and includes subtypes such as adenocarcinoma, squamous cell carcinoma, and large cell carcinoma. SCLC, which comprises around 15% of cases, is more aggressive and rapidly metastasises to distant organs [5]. Despite the advances in cancer treatment, lung cancer remains one of the deadliest cancers, with a five-year survival rate remaining low due to late-stage diagnosis and treatment resistance [6]. Understanding the underlying molecular mechanisms, genetic alterations, and critical pathways involved in lung cancer is crucial for identifying novel therapeutic targets and developing effective treatment strategies. Lung cancer develops through a complex interplay of genetic mutations, environmental factors, and epigenetic modifications [4,7,8]. Carcinogens such as tobacco smoke, air pollution, radon exposure, and occupational hazards contribute significantly to DNA damage, leading to genetic alterations that drive cancer progression [9,10]. These genetic changes result in the activation of oncogenes, inactivation of tumour suppressor genes, and dysregulation of key signalling pathways that control cell proliferation, apoptosis, and differentiation. The disease often progresses silently, with symptoms appearing in the advanced stages, which limits early detection and timely intervention [11,12]. The global burden of lung cancer is staggering. According to recent epidemiological data, approximately 2.2 million new cases of lung cancer are diagnosed annually, making it the second most common cancer worldwide. The mortality rate is alarmingly high, with nearly 1.8 million deaths per year, emphasising the need for improved diagnostics and therapeutic options to combat lung cancer [9,10].

Several genes play a pivotal role in the initiation and progression of lung cancer. These include tumour suppressor genes such as TP53, RB1, and STK11 and oncogenes like KRAS, EGFR, ALK, and MYC [13]. Mutations or aberrant expression of these genes disrupt normal cellular processes, leading to unchecked growth and metastasis. In addition to these well-established players, structural and functional insights into proteins encoded by specific genes provide valuable information for targeted drug design [14]. The following PDB structures correspond to proteins that are intricately linked to lung cancer pathogenesis: Human Cyclin-Dependent Kinase 2 (CDK2) (PDB ID: 1AQ1), Human Protein Kinase CK2 Holoenzyme (PDB ID: 1JWH), Insulin-like Growth Factor 1 Receptor Kinase (PDB ID: 1K3A), Phosphorylated Crk-II (PDB ID: 2DVJ), and Human Aldo-Keto Reductase Family 1 Member B10 (AKR1B10) (PDB ID: 4XZL) [15–19]. These proteins regulate crucial pathways involved in lung cancer and serve as potential drug targets, making them promising candidates for novel therapeutic interventions. Cyclin-dependent kinase 2 (CDK2) is a serine/threonine kinase that plays a crucial role in cell cycle regulation, particularly during the G1 to S phase transition. Dysregulation of CDK2 activity is commonly observed in lung cancer, leading to uncontrolled proliferation. The aberrant activation of CDK2 is often associated with alterations in cyclins and CDK inhibitors such as p21 and p27. Targeting CDK2 has emerged as a promising strategy in cancer therapy to halt cell cycle progression and induce apoptosis in tumour cells [15]. Protein Kinase CK2 is a ubiquitous kinase that regulates cellular processes, including proliferation, apoptosis, and signal transduction. CK2 is frequently overexpressed in lung cancer and contributes to tumour growth by phosphorylating key oncogenic proteins, including AKT, NF-κB, and β-catenin. Inhibiting CK2 activity has sensitised cancer cells to chemotherapeutic agents, making it a viable target for lung cancer therapy [16]. The Insulin-like Growth Factor 1 Receptor (IGF-1R) is a receptor tyrosine kinase that mediates cell growth and survival by activating the PI3K/AKT and RAS/RAF/MEK/ERK pathways. Overexpression and hyperactivation of IGF-1R have been implicated in lung cancer progression and resistance to therapy. Targeting IGF-1R with specific inhibitors or monoclonal antibodies can disrupt cancer cell survival signals and enhance the efficacy of existing treatments [17]. Phosphorylated Crk-II is an adaptor protein involved in signal transduction pathways that regulate cell migration, adhesion, and proliferation [18]. Phosphorylation of Crk-II plays a critical role in lung cancer progression by modulating the activity of downstream signalling molecules. Targeting Crk-II or its interacting partners could potentially suppress metastatic spread in lung cancer patients [18]. Human Aldo-Keto Reductase Family 1 Member B10 (AKR1B10) is an enzyme in lipid metabolism and detoxification, and overexpression has been linked to lung cancer, particularly in smokers [6,19].

Multitargeted drug design (MTDD), or designing the Polypharmacological drug, is a therapeutic strategy to modulate multiple disease-associated targets simultaneously. Unlike traditional single-target drugs, multitargeted agents offer several advantages, including reduced resistance, improved efficacy, and broader therapeutic effects [20–23]. MTDD is particularly valuable in complex diseases like lung cancer, where multiple pathways contribute to tumour growth and survival. Several multitargeted drugs have been explored for lung cancer treatment [24–26]. For example, tyrosine kinase inhibitors (TKIs) such as crizotinib, gefitinib, and osimertinib target multiple oncogenic kinases, providing significant clinical benefits. Rational drug design approaches can be employed to develop inhibitors that simultaneously target CDK2, CK2, IGF-1R, Crk-II, and AKR1B10 in the context of the mentioned PDB structures [14,19]. The development of drugs targeting the aforementioned proteins holds significant promise for lung cancer treatment. Structure-based drug design (SBDD) approaches can leverage the crystal structures of these proteins to identify potent inhibitors with high specificity and minimal off-target effects. Integrating AI-driven drug discovery platforms can further accelerate the identification and optimisation of lead compounds. By designing inhibitors that can simultaneously modulate multiple key targets, researchers can develop more effective therapies that address the complexity of lung cancer. Future research should focus on validating potential inhibitors through preclinical and clinical studies to translate these findings into effective therapies for lung cancer patients. Moreover, integrating predictive modelling with molecular docking and AI-based screening may significantly enhance the efficiency of identifying viable drug candidates. While significant progress has been made in lung cancer research, several challenges remain. [14] Drug resistance, tumour heterogeneity, and the dynamic nature of cancer signalling networks complicate treatment strategies. Developing more effective multitargeted drugs, combined with personalised medicine approaches based on genetic profiling, could revolutionise lung cancer therapy in the coming years [11]. Additionally, integrating immunotherapy with multitargeted approaches holds promise in overcoming resistance and improving patient outcomes [27].

In this study, we performed extensive screening of the DrugBank Library against crucial proteins from lung cancer and identified a Pentapharmacological (5 targets) drug candidate with the least drug resistance capabilities. The study was extended to validate the identified candidate with interaction fingerprints, pharmacokinetics, DFT, WaterMap, MD Simulation, and MM/GBSA.

## 2. Methods

**Fig 1:**
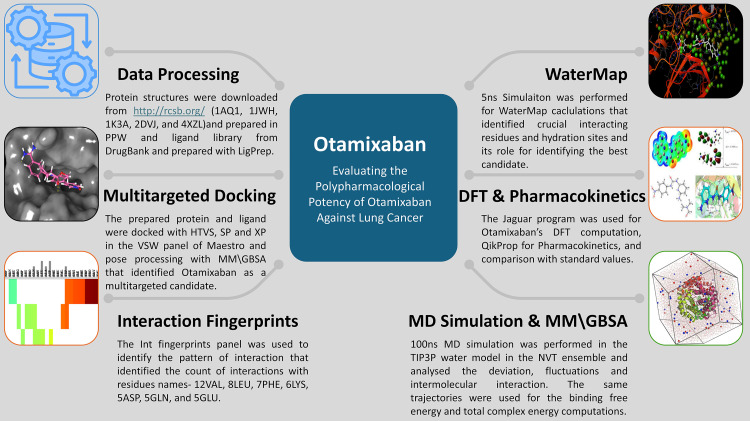
The workflow of the complete study shows the steps followed from data preprocessing to identification and validation of Otamixaban as a Pentapharmacological drug candidate.

We performed several studies in this study, and Fig 1 is plotted to clarify it. Further, the detailed stepwise methods are as follows

### 2.1 Protein and ligand data collection and preparation

Accurate protein preparation is a foundational step in molecular docking and structure-based drug design that involves critical steps, such as removing crystallographic water molecules, adding missing hydrogen atoms, correcting bond orders, properly assigning atomic charges, and subsequent energy minimisation to stabilise the structure [28]. These modifications help resolve incomplete side chains or incorrect geometries, yielding biologically meaningful models suitable for computational simulations. In this study, protein structures involved in lung cancer, including the Human cyclin-dependent kinase-2 (protein kinase, PDBID- 1AQ1), Human protein kinase ck2 holoenzyme (transferase, PDBID- 1JWH), Insulin-like growth factor 1 receptor kinase (transferase, PDBID- 1K3A), Phosphorylated CRK-II (signalling protein, PDBID- 2DVJ), and Human akr1b10 (oxidoreductase, PDBID- 4XZL), were retrieved from the RCSB Protein Data Bank (https://www.rcsb.org/) [15–19,29–31]. The downloaded PDB files consisted of various chains and molecular components such as Chain A, solvents and ligand in 1AQ1, Chain A, B, C and D, and solvents and ligand in 1JWH, Chain A and ligand in 1K3A, Chain A in 2DVJ, and Chain X, solvents and ligand in 4XZL. These files were imported into the Protein Preparation Workflow (PPW) module in Schrödinger Maestro (v.2024–2) for comprehensive preparation [32,33]. The preparation protocol included the capping of termini, reconstruction of missing side chains, assignment of proper bond orders, and generation of disulfide bonds. Zero bond orders were assigned to metal atoms as needed. Missing loop regions were reconstructed using Prime, while the protonation and heteroatom states were predicted using Epik at a physiological pH of 7.4 ± 2—additional steps involved optimising water molecule orientation and minimising the positions of hydrogens on altered species [32,34–36]. The overall protein structures were optimised using PROPKA for pH-dependent state adjustments and subjected to energy minimisation of all atoms with a convergence threshold of 0.30 Å using the OPLS4 force field [32,37,38]. Crystallographic water molecules located beyond 5 Å from any ligand were deleted to improve docking accuracy. Finally, to retain only the relevant portions of the structure for grid generation, Chain A with ligand was preserved in 1AQ1, 1JWH, 1K3A, and Chain X in 4XZL, while only Chain A was preserved in 2DVJ.

Ligand preparation is critical to ensure that small molecules are chemically accurate, stereochemically defined, and energetically minimised before subjecting them to docking or virtual screening. This includes standardising chemical structures by correcting improper bond orders, assigning correct atom types, adding explicit hydrogen atoms, and generating realistic 3D conformers. Optimisation also involves generating relevant ionisation states, tautomeric forms, and stereoisomers under biologically relevant pH conditions. This study obtained a comprehensive DrugBank ligand library (https://go.drugbank.com/) and processed it using the LigPrep tool in Schrödinger Maestro (v.2024−2) [32,39]. Compounds with more than 500 atoms were removed as part of the preprocessing step to eliminate excessively large molecules. The remaining compounds underwent pH-based ionisation and tautomer generation using the classic Epik module, targeting a physiological pH of 7 ± 2 [32,36]. Desalting was performed to remove extraneous counter-ions. Stereoisomer generation was carried out while maintaining defined chiral centres, producing up to 32 stereoisomers per original ligand structure. The final processed ligand set was stored in SDF format and prepared for downstream docking studies.

### 2.2 Receptor grid generation and molecular docking strategies

Receptor grid generation defines the spatial domain within which molecular docking is performed, simulating the environment surrounding the protein’s active site. This involves building a three-dimensional grid around the region of interest—typically around a co-crystallised ligand or known active residues—to encompass key interaction sites. In this study, grids were constructed on all protein structures to facilitate blind docking and enable the identification of the most appropriate multitarget ligand. Grid generation was conducted using the Receptor Grid Generation tool in Schrödinger Maestro (v.2024–2) [32,40]. Parameters were adjusted by setting a scaling factor of 1 and a partial charge cutoff of 0.25. The site was defined to include all residues, and the grid box was centred to span the entire protein structure. The “Dock ligands with length” setting was optimised to ensure the proper fit of the molecules, and the resulting grid files were saved in compressed zip format. The multitargeted docking strategy used in this study aimed to identify ligands capable of binding simultaneously to multiple protein targets. This involved individually generating receptor grids for each protein, tailored to their respective active sites and binding environments. The prepared ligand library was then introduced into Schrödinger’s Virtual Screening Workflow (VSW), where we opted to generate unique properties per unique compound to prevent duplicate screening and conserve computational resources [32,41]. The pharmacokinetic screening was conducted using QikProp to calculate relevant molecular descriptors, and compounds were filtered based on Lipinski’s rule of five to enhance drug-likeness [27,32,42–45]. Within the receptor tab of the VSW module, individual receptor grids were loaded one at a time. In the docking tab, advanced options such as enhanced planarity enforcement for conjugated π-systems and application of Epik state penalties were activated [32,36]. The van der Waals interactions scaling factor was set to 0.80, and the partial charge cutoff was adjusted to 0.15. These modifications help manage steric clashes and filter insignificant polar contacts, refining docking accuracy. A tiered docking approach was implemented: high-throughput virtual screening (HTVS) for rapid screening, standard precision (SP) for intermediate filtering, and extra precision (XP) docking for in-depth interaction analysis. The docking results were further subjected to post-docking rescoring using MM/GBSA (molecular mechanics combined with generalised Born and surface area solvation), which was applied to each docking pose to estimate binding free energy. The entire ligand set passed through successive filtering to HTVS, the top 10% from HTVS to SP, the top 50% from SP to XP, and 100% of XP docking poses were subjected to MM/GBSA analysis. These results were exported in CSV format for systematic evaluation, allowing for the identification of the most promising multitarget ligands based on their docking performance.

### 2.3 Molecular interaction fingerprint generation

Molecular Interaction Fingerprints (MIFs) provide a systematic and computationally efficient method for analysing and representing the complex non-covalent interactions between a ligand and its target protein. MIFs reduce intricate interaction data into simplified binary or numerical vectors, capturing essential interaction types such as hydrogen bonds, hydrophobic contacts, π-π stacking, van der Waals forces, and ionic interactions. These fingerprints are valid for comparative analysis of ligand binding profiles across multiple targets or conformations. The Interaction Fingerprint module in Schrödinger Maestro (v.2024−2) was employed for this analysis. Protein-ligand complexes for all five target proteins were selected, and the option to focus on receptor-ligand interactions was enabled. Alignment was performed using PDB ID 1AQ1 as the reference structure to standardise the comparison. The generated interaction matrix captured all relevant residue-level contacts. A colour-coded plot visualised interaction patterns, with the protein marked N- to C-terminal progression. Only residues with meaningful ligand interactions were retained for subsequent analysis, and the frequency of such interactions was tabulated to highlight key binding determinants.

### 2.4 WaterMap calculations

WaterMap is a specialised computational approach to characterise water molecules’ thermodynamic landscape surrounding a protein’s binding site. It identifies hydration sites that significantly contribute to ligand binding affinity, distinguishing between favourable and unfavourable water molecules [46]. This study used the Schrödinger WaterMap calculations panel that uses Desmond in the background to compute it on docked protein-ligand complexes. The region within 10 Å of the docked ligand was selected for water analysis [32,47,48]. The simulation setup involved truncation of the protein structure, application of the OPLS4 force field, and designation of existing water molecules as solvents [32,37]. The simulation time was fixed at 5 ns, and trajectory files were not generated to streamline the analysis. Following the simulation, results were interpreted using Schrödinger Maestro’s WaterMap Examine Results panel. The workspace was analysed to identify key hydration features such as enthalpy, entropy, free energy, and overlap factor of water clusters [32,47,48]. These data points allowed assessment of the displacement or retention of water molecules during ligand optimisation, offering insights into water-mediated interactions crucial for improving binding affinity and specificity.

### 2.5 DFT and pharmacokinetics of the identified drug candidates

Density Functional Theory (DFT) is a quantum mechanical method for investigating chemical compounds’ electronic structures and properties. It provides information about molecular reactivity, charge distribution, dipole moments, and HOMO-LUMO energy gaps—all critical parameters in assessing ligand stability and activity [49]. For this work, DFT simulations were executed through the Optimisation panel in Schrödinger Maestro (v.2024−2), utilising the Jaguar program [32,50,51]. The B3LYP-D3 exchange-correlation functional was used with the 6-31G** basis set to obtain accurate molecular geometries and energetics predictions [52]. Automatic SCF spin treatments and atomic overlap options were used for SCF convergence [32,53]. A maximum of 48 iterations was permitted, with an energy threshold set at 5e-05 Hartree. The geometry optimisation involved 100 steps under default convergence criteria. Quantum properties computed included HOMO, LUMO, and molecular orbital surfaces. Solvent effects were modelled using the PBF model for water [32,54]. Results were monitored through the QM convergence panel, and output files were archived for further interpretation [32,50,51]. Pharmacokinetic analysis focused on ADME properties, including absorption, distribution, metabolism, and excretion. Schrödinger QikProp was used to generate molecular descriptors related to these processes, and results were evaluated based on Lipinski’s rule of five [32,44,45]. Comparisons were made with standard benchmarks within QikProp to identify compounds with optimal drug-like properties [55].

### 2.6 MD simulation and binding free energy calculations

Molecular Dynamics (MD) Simulation enables the exploration of biomolecular behaviour over time, revealing dynamic aspects of protein-ligand interactions that static models cannot capture. In this work, MD simulations were carried out using the Desmond module (https://www.deshawresearch.com/) in Schrödinger Maestro (v.2024−2) for five protein-ligand complexes [32,47,48]. The simulation process was divided into three stages. The first stage involved preparing the system using the System Builder, where the TIP3P water model was used to solvate the protein-ligand complex within a 10 × 10 × 10 Å buffer box [32,56 ]. The box size was optimised for minimum volume, and visual checks ensured the complex was adequately encapsulated. Electrostatic neutrality was achieved by introducing counter-ions 6Cl^-^ 1AQ1 and 1JWH, 16 Na^+^ in 1K3A, 2Na^+^ in 2DVJ and 12 Na^+^ in 4XZL complex, and OPLS4 force field was used [32,37]. In the second stage, the prepared systems were subjected to a 100 ns production run using the Molecular Dynamics module in Schrödinger Maestro. Frame capture intervals were set at 100 ps, generating 1000 frames per simulation. The system was maintained under NPT conditions (300 K, 1.01 atm) and equilibrated before production [32,57]. In the third stage, trajectory analysis was conducted to evaluate structural deviations, flexibility, and interaction stability. Key metrics such as root-mean-square deviation (RMSD), root-mean-square fluctuation (RMSF), and hydrogen bonding profiles were computed. Graphical plots were generated to visualise temporal behaviour and assess the consistency of binding modes.

Subsequently, MM/GBSA computations were performed on selected snapshots from the MD trajectories. This method estimated the binding free energy using van der Waals, electrostatic, and solvation contributions [58]. The following bash command was used to run the energy calculations:

First, we exported the Schrödinger in the terminal to access it-
  export SCHRODINGER=/opt/Schrodinger-VERSION/
and then run kept to run the main job with the thermal_mmgbsa.py file
  $SCHRODINGER/run thermal_mmgbsa.py desmond_NAME-out.cms

The resulting energy profiles provided insights into each protein-ligand complex’s thermodynamic stability and binding strength, validating docking predictions and assisting in lead optimisation.

## 3. Results

### 3.1 Protein structure validation and optimisation

**Fig 2:**
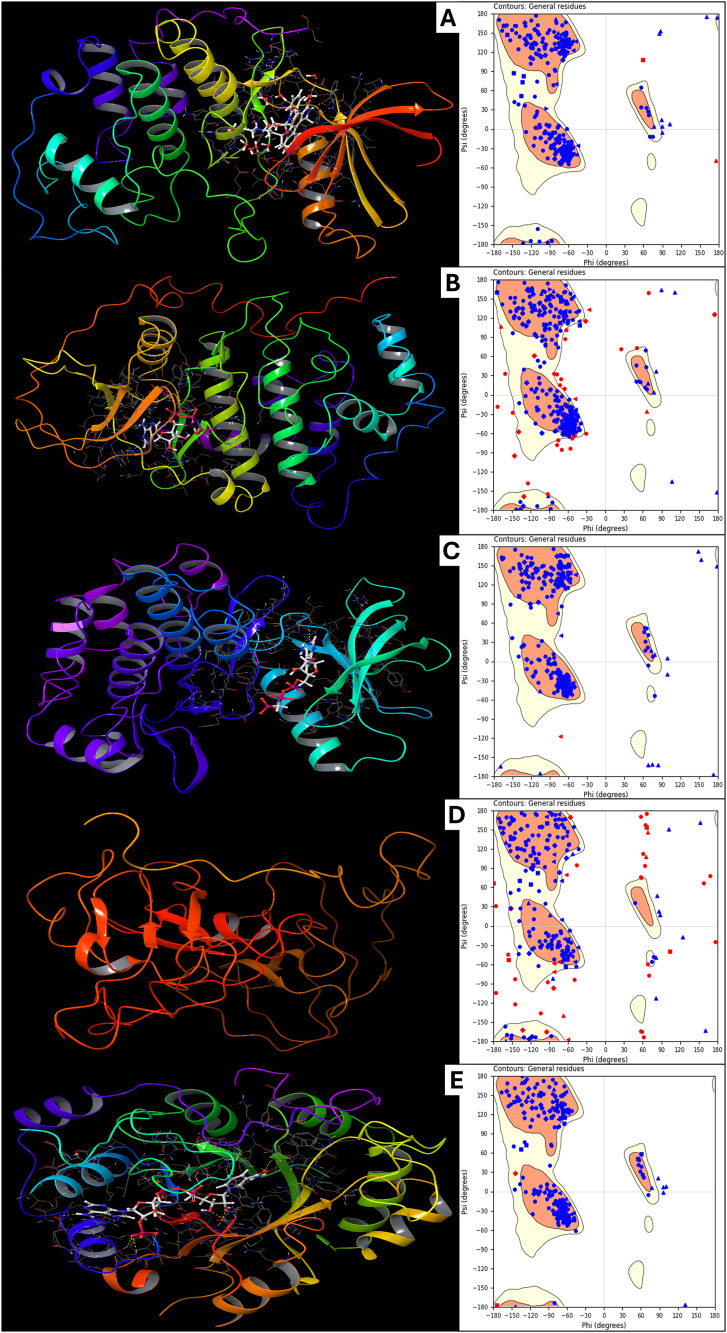
Showing the Prepared Protein Structures and Ligand Binding Sites and respective Ramachandran Plot for A) Human cyclin-dependent kinase-2 (protein kinase, PDBID- 1AQ1), B) Human protein kinase CK2 holoenzyme (transferase, PDBID- 1JWH), C) Insulin-like growth factor 1 receptor kinase (transferase, PDBID- 1K3A), D) Phosphorylated CRK-II (signalling protein, PDBID- 2DVJ), and E) Human akr1b10 (oxidoreductase, PDBID- 4XZL).

The protein preparation reports generated using the Protein Preparation Workflow in Maestro for five lung cancer-related proteins provide insight into their structural stability and energy characteristics. The proteins analysed include Human CDK2 (1AQ1), Human Protein Kinase CK2 Holoenzyme (1JWH), Insulin-like Growth Factor 1 Receptor Kinase (1K3A), phosphorylated Crk-II (2DVJ), and human AKR1B10 (4XZL). The detailed energy components of each system have been evaluated to understand the energetic contributions that govern protein stability and function. For 1AQ1, the CDK2, the system’s total energy and potential energy are recorded at −1.45E + 03 kcal/mol, indicating a stable system with a well-minimised structure. The bond stretch energy is 1.55E + 02 kcal/mol, which suggests minor structural strain within covalent bonds. The angle bending and torsion angle energies are 7.15E + 02 kcal/mol and 5.55E + 02 kcal/mol, respectively, reflecting the flexibility required for functional conformational changes. The Lennard-Jones energy accounts for van der Waals interactions and is −3.02E + 03 kcal/mol, indicating strong non-covalent interactions within the protein structure. Electrostatic energy contributes −1.84E + 03 kcal/mol, highlighting the importance of charge-based interactions in stabilising the protein fold. The absence of hydrogen bond energy suggests that explicit hydrogen bonding contributions may be implicitly included in other electrostatic interactions. For 1JWH, the Human Protein Kinase CK2 Holoenzyme, the total system energy is −3.08E + 03 kcal/mol, making it the most stabilised system among the analysed proteins. The high bond stretch (4.95E + 02 kcal/mol), angle bending (2.55E + 03 kcal/mol), and torsion energy (2.31E + 03 kcal/mol) indicate that this kinase has a structurally constrained yet flexible conformation necessary for enzymatic function. The 1,4 Lennard-Jones energy is 5.46E + 03 kcal/mol, suggesting strong short-range interactions between side chains. The total electrostatic energy is −5.62E + 03 kcal/mol, implying significant stabilisation from charge-based interactions. This kinase’s structure is highly influenced by van der Waals and electrostatic interactions, which are essential for its catalytic activity and regulatory function. For 1K3A, the Insulin-like Growth Factor 1 Receptor Kinase, the total energy is −2.06E + 03 kcal/mol, reflecting a stable protein fold. The bond stretch energy (1.51E + 02 kcal/mol), angle bending energy (6.55E + 02 kcal/mol), and torsion angle energy (5.55E + 02 kcal/mol) are moderate, indicating a balanced structural conformation. The Lennard-Jones energy is −3.26E + 03 kcal/mol, suggesting effective packing and strong non-covalent stabilisation. Electrostatic energy contributes −2.07E + 03 kcal/mol, reinforcing the significance of charge-based interactions in kinase stability. The balance between van der Waals forces and electrostatic stabilisation ensures the correct alignment of catalytic residues in the active site. For 2DVJ, phosphorylated Crk-II, the total energy is recorded as 4.73E + 02 kcal/mol, notably different from the other proteins, indicating a relatively higher energy, possibly a less stable conformation. The bond stretch energy (1.06E + 02 kcal/mol), angle bending energy (6.03E + 02 kcal/mol), and torsion angle energy (7.35E + 02 kcal/mol) suggest that this protein has significant structural flexibility. The Lennard-Jones energy is −1.59E + 03 kcal/mol, and the electrostatic energy is −8.65E + 02 kcal/mol, indicating moderate contributions to protein stability. Given that Crk-II undergoes phosphorylation-dependent conformational changes, these energetic values may reflect an intermediate structural state rather than a fully stable fold. For 4XZL, human AKR1B10, the system’s total energy is −2.22E + 03 kcal/mol, indicating a relatively stable conformation. The bond stretch energy (1.70E + 02 kcal/mol), angle bending energy (7.54E + 02 kcal/mol), and torsion angle energy (5.25E + 02 kcal/mol) reveal a structurally constrained but functionally adaptable protein. The Lennard-Jones energy is −3.60E + 03 kcal/mol, reflecting strong van der Waals interactions that aid in maintaining the protein’s tertiary structure. Electrostatic energy is −2.32E + 03 kcal/mol, supporting the role of charge-based interactions in protein stability. The interplay of these energetic components ensures the functional integrity of AKR1B10 in cellular redox processes. The energy contributions from bond stretching, angle bending, and torsion angles highlight each protein structure’s intrinsic flexibility and strain, which is necessary for its respective functions. The Lennard-Jones interactions play a crucial role in maintaining the compactness of the proteins, preventing destabilising clashes while ensuring proper packing of amino acid side chains. Electrostatic interactions significantly contribute to the overall stability, particularly in kinases and enzymes, where charge distribution is critical for activity. The variation in total energy values among the proteins suggests differences in their intrinsic stability, which can be correlated with their biological functions and interaction partners. The relatively higher energy value for phosphorylated Crk-II indicates that post-translational modifications like phosphorylation can increase conformational flexibility, which is crucial for signalling proteins. These energetic parameters provide insights into the stability, conformational preferences, and potential dynamic behaviour of these lung cancer-associated proteins, which can be useful for structure-based drug design and functional studies. Further, Fig 2 better represents the 3D structures of prepared proteins with their Ramachandran Plots to make it clearer.

### 3.2 Molecular docking studies

**Fig 3:**
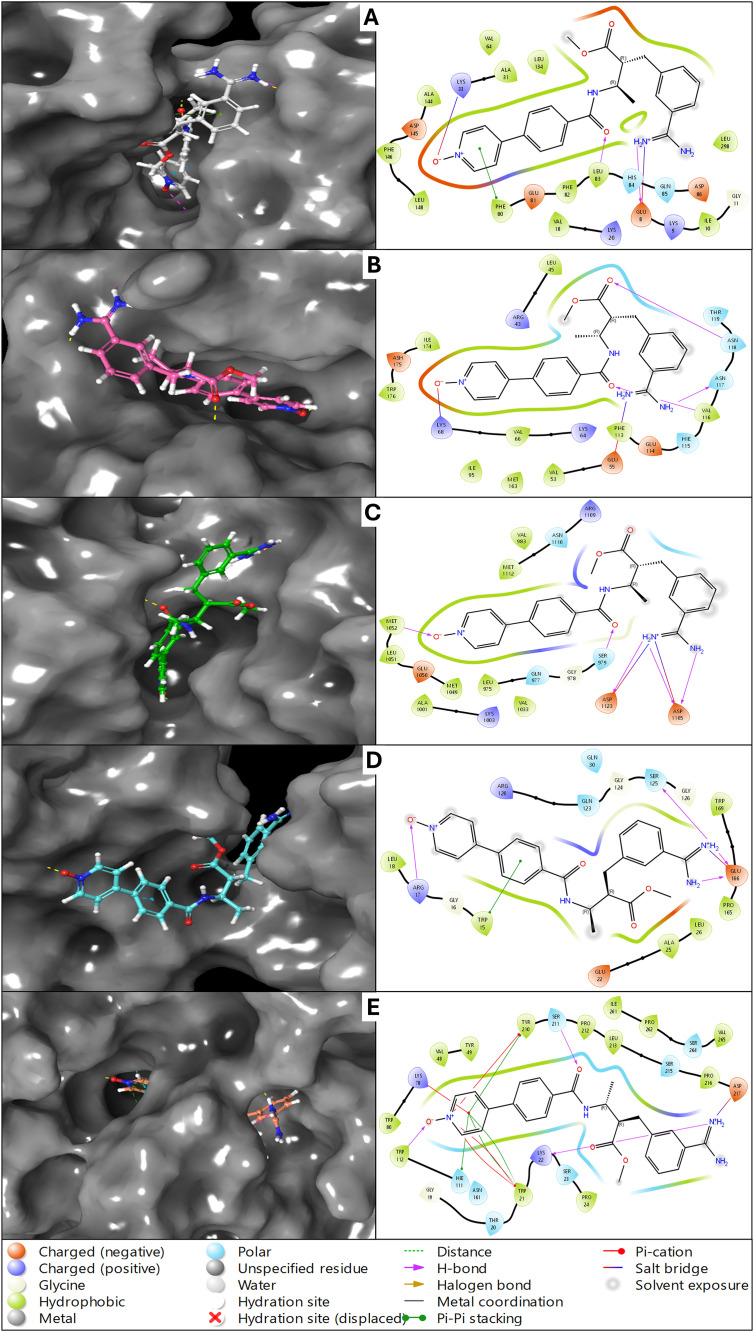
Showing the Docked poses in 3D and 2D as Ligand Interaction Diagram for A) Human cyclin-dependent kinase-2 (protein kinase, PDBID- 1AQ1), B) Human protein kinase CK2 holoenzyme (transferase, PDBID- 1JWH), C) Insulin-like growth factor 1 receptor kinase (transferase, PDBID- 1K3A), D) Phosphorylated CRK-II (signalling protein, PDBID- 2DVJ), and E) Human akr1b10 (oxidoreductase, PDBID- 4XZL) in complex with Otamixaban and the legend is shown for identifying the bond and residue types.

The multitargeted molecular docking has resulted in very promising results. However, Otamixaban has results as the most promising drug candidate, showing the top scores in all five protein cases, and we have analysed them in detail. The interactions regarding the residues, their chemical nature, and the bond types facilitating the ligand binding are detailed. Otamixaban interacts with the ATP-binding site of CDK2, forming multiple stabilising interactions. The ligand establishes hydrogen bonds with Lys33 (positively charged) and Glu81 (negatively charged), ensuring a strong polar anchoring within the active site. Hydrophobic interactions are observed with Phe80, Leu83, and Val64, which provide structural stability through van der Waals forces. Notably, a Pi-cation interaction is formed with Lys33, further stabilising the ligand in the active pocket. Electrostatic interactions are also present between Otamixaban and Asp145, reinforcing binding stability. Solvent exposure of the ligand’s polar groups suggests partial accessibility to water molecules, which may influence ligand flexibility and binding dynamics (Fig 3). In the case of CK2, Otamixaban establishes key hydrogen bonds with Lys68, Glu81, and Asp175, which are critical residues within the active site. A strong salt bridge interaction is noted with Glu114, enhancing the ligand’s anchoring within the catalytic pocket. The ligand also engages in hydrophobic interactions with residues such as Leu63, Val64, and Ile174, which contribute to the stabilisation of Otamixaban in the hydrophobic regions of CK2. Pi-Pi stacking interactions with Phe80 suggest an additional stabilising factor, promoting the favourable orientation of the ligand. Solvent exposure of the ligand’s polar regions indicates potential interactions with surrounding water molecules, which might play a role in ligand dynamics and bioavailability (Fig 3). Otamixaban binds within the kinase domain of IGF-1R through a series of hydrogen bonds with Asp1123, Glu1081, and Lys1030, which are functionally important residues involved in ATP coordination. Hydrophobic interactions are formed with Leu1091, Met1078, and Val1055, providing a stabilising environment for ligand accommodation. A Pi-cation interaction is observed with Lys1030, strengthening the ligand’s positioning within the active site. Electrostatic interactions with Asp1123 further contribute to ligand stability, possibly enhancing its affinity for IGF-1R. The hydration site displacement suggests a rearrangement of water molecules upon ligand binding, optimising receptor conformation for Otamixaban interaction (Fig 3). Otamixaban exhibits distinct binding interactions within phosphorylated Crk-II. The ligand forms multiple hydrogen bonds with Gly121, Ser122, and Arg124, which are crucial residues in the recognition loop of Crk-II. Hydrophobic interactions with Leu107 and Ile119 support the ligand’s orientation within the binding cavity. A notable salt bridge interaction occurs with Glu126, further stabilising the ligand’s position. A halogen bond with Ser122 suggests an additional interaction mechanism, reinforcing ligand binding. Pi-Pi stacking interactions with Trp115 may contribute to ligand stability by promoting aromatic interactions (Fig 3). The structural arrangement indicates that Otamixaban may influence the conformational dynamics of Crk-II upon binding. A combination of hydrogen bonding, hydrophobic contacts, and Pi-Pi stacking interactions characterises the interaction between Otamixaban and AKR1B10. Hydrogen bonds are formed with Ser46, Glu49, and Asn167, contributing to ligand stabilisation. Hydrophobic interactions involving Trp112, Phe123, and Leu125 support the ligand’s positioning within the binding site. A Pi-cation interaction is observed with Arg24, stabilising the ligand’s orientation. Additionally, the ligand forms a salt bridge with Glu49, further enhancing its binding affinity. The presence of solvent exposure in certain regions of the ligand suggests that water-mediated interactions might play a role in modulating Otamixaban’s stability within AKR1B10 (Fig 3).

The docking analysis of Otamixaban against five different lung cancer-related proteins—CDK2 (1AQ1), Protein Kinase CK2 (1JWH), IGF-1R Kinase (1K3A), phosphorylated Crk-II (2DVJ), and AKR1B10 (4XZL)—revealed varying binding affinities and interaction patterns as shown in Table 1. Among these, the CDK2 complex (1AQ1) demonstrated the highest binding affinity, with a docking score of −11.841 and an MM/GBSA dG Bind of −69.96 kcal/mol, indicating strong thermodynamic stability. This was followed by AKR1B10 (4XZL) with a docking score of −10.246 and binding free energy of −65.51 kcal/mol, suggesting a similarly strong binding interaction. Notably, 1AQ1 and 4XZL exhibited high hydrogen bonding contributions (−2.023 and −2.095, respectively), indicating critical polar interactions stabilising the ligand. On the other hand, Protein Kinase CK2 (1JWH) and IGF-1R Kinase (1K3A) showed moderate docking scores (−8.381 and −6.52, respectively), with lower hydrogen bond contributions (−0.9 and −1.52, respectively). However, MM/GBSA dG Bind values (−46.87 for 1JWH and −45.22 for 1K3A) suggest moderate binding stability. Phosphorylated Crk-II (2DVJ) had the lowest docking score (−6.541), with a binding free energy of −49.00 kcal/mol, suggesting a comparatively weaker interaction. In terms of complex stability, CDK2 (1AQ1) showed the lowest complex energy (−11433.383), followed by CK2 (1JWH, −13378.059) and IGF-1R (1K3A, −12443.133), indicating strong receptor-ligand interaction stability. Interestingly, the highest receptor hydrogen bonding contribution was observed in CK2 (1JWH, −174.276), followed by IGF-1R (−164.625) and CDK2 (−142.562), highlighting significant structural interactions as shown in Table 1. The docking analysis of Otamixaban with these five lung cancer-related proteins highlights its diverse interaction profile, involving a combination of hydrogen bonding, hydrophobic interactions, electrostatic contacts, and Pi-Pi stacking interactions. The hydrogen bonds contribute significantly to ligand stabilisation by anchoring Otamixaban to key functional residues, while hydrophobic interactions help position the ligand within nonpolar pockets, enhancing its affinity. Electrostatic interactions, including salt bridges with negatively charged residues (e.g., Glu and Asp), further strengthen ligand binding, particularly in proteins like CK2 and AKR1B10. Additionally, Pi-Pi stacking and Pi-cation interactions with aromatic residues such as Phe and Trp play a crucial role in stabilising the ligand-receptor complex. The hydration sites observed in some cases suggest that water molecules may be involved in modulating ligand flexibility and receptor adaptation, potentially influencing the binding affinity and bioavailability of Otamixaban. The variation in interaction patterns across different proteins suggests that Otamixaban could exhibit differential binding affinities and functional consequences, which may be relevant for its potential as a multitargeted therapeutic agent. The results suggest that Otamixaban binds strongly to CDK2 and AKR1B10, making them potential high-affinity targets. The relatively lower binding scores and interaction energies for IGF-1R, CK2, and Crk-II indicate moderate to weak binding, which could affect ligand efficacy in these targets. These results provide valuable insights into the potential multitarget binding capability of Otamixaban, with CDK2 and AKR1B10 being the most promising targets.

**Table 1:** Showing the Docking and other scores produced during the multitargeted molecular docking on Lung Cancer for Otamixaban in complex with Human cyclin-dependent kinase-2 (protein kinase, PDBID- 1AQ1), Human protein kinase CK2 holoenzyme (transferase, PDBID- 1JWH), Insulin-like growth factor 1 receptor kinase (transferase, PDBID- 1K3A), Phosphorylated CRK-II (signalling protein, PDBID- 2DVJ), and Human akr1b10 (oxidoreductase, PDBID- 4XZL).

PDB ID	State Penalty	Docking Score	ligand efficiency sa	XP HBond	MM/GBSA dG Bind
1AQ1	0.0001	−11.841	−3.18	−2.023	−69.96
1JWH	0.0001	−8.381	−2.13	−0.9	−46.87
1K3A	0.0001	−6.52	−2.056	−1.52	−45.22
2DVJ	0.0001	−6.541	−2.227	−1.714	−49
4XZL	0.0001	−10.246	−2.978	−2.095	−65.51
PDB ID	**Complex Energy**	**XP GScore**	**ligand efficiency ln**	**Receptor Hbond**	**MM/GBSA dG Bind Hbond**
1AQ1	−11433.383	−11.841	−15.558	−142.562	−2.26
1JWH	−13378.059	−8.381	−10.423	−174.276	−3.63
1K3A	−12443.133	−6.52	−10.057	−164.625	−3.55
2DVJ	−7098.784	−6.541	−10.898	−60.145	−3.09
4XZL	−10206.317	−10.246	−14.568	−138.287	−2.77

### 3.3 Analysis of molecular interaction fingerprint

**Fig 4:**
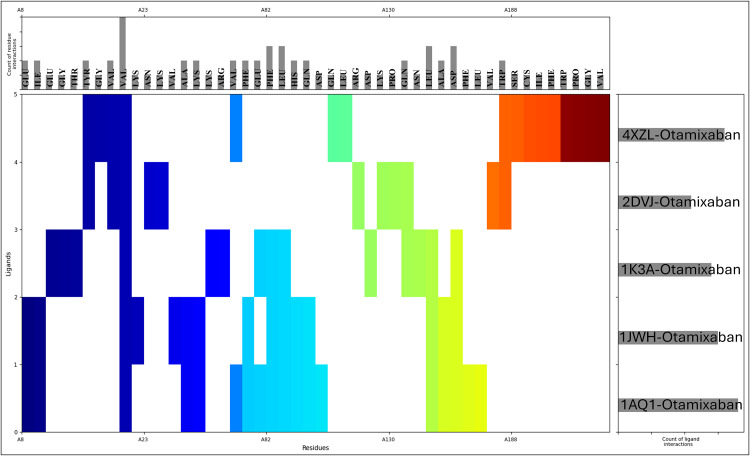
Showing the Molecular Interaction Fingerprints (MIFs) computed on docked poses of Human cyclin-dependent kinase-2 (protein kinase, PDBID- 1AQ1), Human protein kinase CK2 holoenzyme (transferase, PDBID- 1JWH), Insulin-like growth factor 1 receptor kinase (transferase, PDBID- 1K3A), Phosphorylated CRK-II (signalling protein, PDBID- 2DVJ), and Human akr1b10 (oxidoreductase, PDBID- 4XZL) in complex with Otamixaban and the top bar shows the count of interacting residues and right graph shows the counts of ligand interactions, whereas main plot is shown for N to C terminal.

The molecular interaction fingerprint analysis has identified a set of crucial amino acid residues that play a significant role in the binding of Otamixaban to the five target proteins. Valine (VAL) was the most frequently involved residue, appearing 12 times across the docking interactions. As a hydrophobic and nonpolar amino acid, valine primarily contributes to hydrophobic interactions within the binding pocket, stabilising the ligand through van der Waals forces. Its high frequency suggests that Otamixaban predominantly interacts with the proteins’ hydrophobic regions, reinforcing the ligand-binding interface’s nonpolar nature. Leucine (LEU) appeared 8 times in the interaction profiles following valine. Like valine, leucine is a hydrophobic, aliphatic residue that strengthens the hydrophobic core of the ligand-protein complex. Its side-chain interactions contribute to stabilising the binding pocket by providing a nonpolar environment that supports ligand affinity. Similarly, phenylalanine (PHE), observed 7 times, is another hydrophobic residue that engages in pi-stacking interactions with the aromatic rings of Otamixaban, enhancing ligand binding through non-covalent stabilising forces. Lysine (LYS) was present 6 times, indicating a key role in electrostatic interactions (Fig 4). As a positively charged (basic) residue, lysine is frequently involved in salt bridge formation and hydrogen bonding with the ligand, particularly in cases where Otamixaban carries electronegative functional groups. The presence of charged residues like lysine suggests that Otamixaban engages in polar interactions to complement its hydrophobic contacts, making the interaction profile more diverse. Aspartic acid (ASP) and glutamic acid (GLU), both negatively charged acidic residues, were found 5 times each. These residues primarily contribute to electrostatic interactions by forming salt bridges with positively charged functional groups on Otamixaban (Fig 4). They also participate in hydrogen bonding, which enhances the ligand’s specificity toward the binding pocket. Their presence further confirms that Otamixaban balances electrostatic and hydrophobic interactions, which are essential for high-affinity binding. Glutamine (GLN), appearing 5 times, is a polar neutral residue that significantly contributes to hydrogen bonding interactions, which play a key role in ligand stabilisation. The repeated presence of alanine (ALA, 4 occurrences), another hydrophobic residue, reinforces the importance of nonpolar interactions in stabilising Otamixaban within the active sites of the proteins. Glycine (GLY), isoleucine (ILE), and tryptophan (TRP) were each identified 3 times, indicating a mix of hydrophobic stabilisation (ILE, TRP) and structural flexibility (GLY). Due to its small side chain, Glycine provides backbone flexibility, allowing the ligand to be accommodated within the binding pocket without steric hindrance. Tryptophan, an aromatic residue, further enhances pi-pi interactions and contributes to the overall stability of the ligand-protein complex. Arginine (ARG, 2 occurrences), a positively charged residue, further enhances electrostatic interactions, while asparagine (ASN, 2 times) contributes to hydrogen bonding through its amide group. Depending on the local environment, histidine (HIS, 2 times) suggests possible metal coordination or protonated hydrogen bonding. Proline (PRO, 2 times) and Tyrosine (TYR, 2 times) contribute uniquely to binding stability (Fig 4). Proline, with its cyclic structure, often plays a role in restricting backbone flexibility, thereby enhancing structural rigidity in binding regions. With its aromatic and polar hydroxyl group, Tyrosine engages in hydrogen bonding and pi-pi interactions, further stabilising the ligand. The less frequent residues—cysteine (CYS, 1 time), serine (SER, 1 time), and threonine (THR, 1 time)—suggest localised covalent or hydrogen bond interactions that may fine-tune the binding affinity of Otamixaban. Cysteine, in particular, might be involved in disulfide-related interactions or weak van der Waals forces. The repetition of residues across multiple interactions underscores their crucial role in stabilising Otamixaban within the active sites of different proteins. The prevalence of hydrophobic residues (VAL, LEU, PHE, ILE, ALA, TRP) suggests that Otamixaban preferentially binds within hydrophobic pockets, while the presence of polar and charged residues (LYS, ASP, GLU, ARG, GLN, HIS, TYR) indicates additional hydrogen bonding and electrostatic interactions that enhance specificity and affinity (Fig 4). This balance of hydrophobic stabilisation and polar interactions is critical in determining the binding efficiency and selectivity of Otamixaban across different lung cancer-related proteins.

### 3.4 Analysis of WaterMap

**Fig 5:**
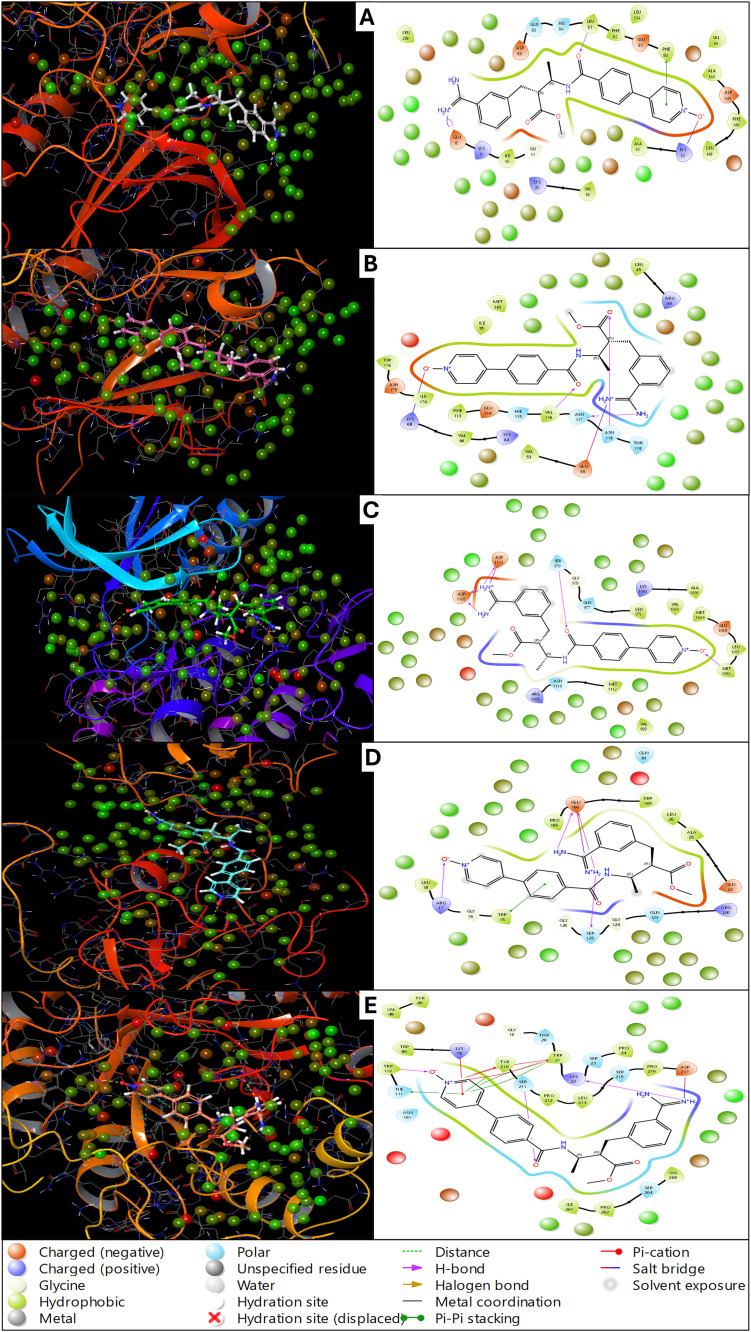
Showing the WaterMap computations in 3D and 2D as Ligand Interaction Diagram for A) Human cyclin-dependent kinase-2 (protein kinase, PDBID- 1AQ1), B) Human protein kinase CK2 holoenzyme (transferase, PDBID- 1JWH), C) Insulin-like growth factor 1 receptor kinase (transferase, PDBID- 1K3A), D) Phosphorylated CRK-II (signalling protein, PDBID- 2DVJ), and E) Human akr1b10 (oxidoreductase, PDBID- 4XZL) in complex with Otamixaban and the legend is shown for identifying the bond and residue types, and hydration sites.

The WaterMap analysis of Otamixaban in complex with five different protein targets—Human CDK2 (1AQ1), Human Protein Kinase CK2 Holoenzyme (1JWH), Insulin-like Growth Factor 1 Receptor Kinase (1K3A), Phosphorylated Crk-II (2DVJ), and Human AKR1B10 (4XZL)—reveals a highly dynamic interplay between hydration sites, water displacement, and direct molecular interactions that govern ligand binding efficiency. Each target exhibits a unique hydration environment, influencing Otamixaban’s binding affinity and stability through water-mediated hydrogen bonding, salt bridges, and hydrophobic interactions. In the case of CDK2 (1AQ1), Otamixaban’s binding is characterised by the displacement of several high-energy water molecules within the active site, particularly near key residues such as Lys33, Glu81, and Asp86. Removing these structured water molecules allows for the formation of direct hydrogen bonds, enhancing ligand stability. The interactions between Otamixaban and Asp145 and Lys89 are particularly strong, contributing to its binding affinity. Additionally, pi-stacking interactions with Phe82 stabilise the ligand further, while forming a salt bridge with Lys33 adds electrostatic stabilisation. The WaterMap analysis suggests that the energetic favourability of displacing these hydration sites plays a critical role in Otamixaban’s affinity for CDK2, reinforcing its potential as a kinase inhibitor (Fig 5). Similarly, in the case of Human Protein Kinase CK2 Holoenzyme (1JWH), hydration site displacement is observed near Asp175, Lys68, and Glu81, where structured water molecules previously facilitated polar contacts. Otamixaban efficiently replaces these hydration sites with direct hydrogen bonds, particularly Asp175, Glu81, and Lys68, strengthening its interactions within the binding pocket. The phenyl ring of Phe113 contributes to pi-stacking interactions, further stabilising the ligand. A strong salt bridge with Arg47 enhances electrostatic complementarity, leading to stronger binding affinity (Fig 5). WaterMap data for CK2 confirms that displacing unfavourable hydration sites while forming strong direct interactions is a key determinant of ligand binding. For the Insulin-like Growth Factor 1 Receptor Kinase (1K3A), the WaterMap analysis reveals a more nuanced role of hydration, where both displacement and retention of water molecules contribute to Otamixaban’s binding efficiency. Several high-energy water molecules near Asp1163, Glu1148, and Lys1150 are displaced, allowing for the formation of direct hydrogen bonds with the ligand. However, some hydration sites remain occupied, suggesting that retained water molecules play an active role in stabilising the ligand. Direct interactions between Otamixaban and Glu1148 and Lys1150 are particularly strong, further supported by pi-stacking interactions with Trp1157. A salt bridge with Asp1163 reinforces the ligand’s binding stability, making IGF-1R a favourable target (Fig 5). WaterMap findings suggest that Otamixaban’s ability to balance hydration site displacement while retaining some stabilising water-mediated interactions is crucial for its high-affinity binding. In the case of phosphorylated Crk-II (2DVJ), hydration plays a slightly different role than other proteins. Unlike CDK2 and CK2, where hydration site displacement is dominant, Crk-II retains many structured water molecules that actively contribute to ligand stability. WaterMap analysis indicates that Otamixaban displaces hydration sites near Asp39, Arg40, and Glu87, allowing for stronger direct hydrogen bonds. However, several hydration sites remain occupied, suggesting that water molecules facilitate rather than hinder ligand binding. Key hydrogen bonds with Glu87 and Asn88 contribute significantly to ligand stabilisation. Additionally, the presence of coordinated metal ions, possibly zinc or magnesium, near the binding pocket may further influence the ligand’s binding efficiency. A salt bridge between Otamixaban and Asp39 and Arg40 enhances electrostatic interactions, reinforcing the stability of the ligand-protein complex. Unlike other targets, Crk-II utilises water molecules as a stabilising factor rather than relying solely on hydration site displacement (Fig 5). Finally, in the case of Human AKR1B10 (4XZL), Otamixaban exhibits a highly specific pattern of hydration site displacement. Several high-energy water molecules near Asp43, Tyr50, and Ser48 are displaced, allowing for the formation of direct interactions with the protein. However, a few hydration sites near Glu54 remain occupied, indicating that water-mediated hydrogen bonding still plays a role in stabilising the ligand. Direct hydrogen bond interactions with Ser48, Asp43, and Glu54 reinforce Otamixaban’s affinity for AKR1B10. Additionally, a strong pi-cation interaction with Lys77 further stabilises the ligand, while a crucial salt bridge with Glu54 enhances electrostatic attraction. (Fig 5) WaterMap analysis reveals that Otamixaban’s ability to selectively displace hydration sites while retaining key stabilising water molecules is essential for optimising its binding efficiency to AKR1B10. WaterMap analysis of Otamixaban across these five protein targets underscores hydration’s complex and context-dependent role in ligand binding. While targets such as CDK2 and CK2 exhibit significant hydration site displacement, facilitating strong direct interactions, proteins like Crk-II rely more on retained water molecules to stabilise ligand binding (Fig 5). The findings highlight that Otamixaban’s binding affinity is not solely dictated by direct protein-ligand interactions but also by the strategic displacement and retention of hydration sites, shaping its specificity and stability on protein targets.

### 3.5 Analysis of DFT and pharmacokinetics results

**Fig 6:**
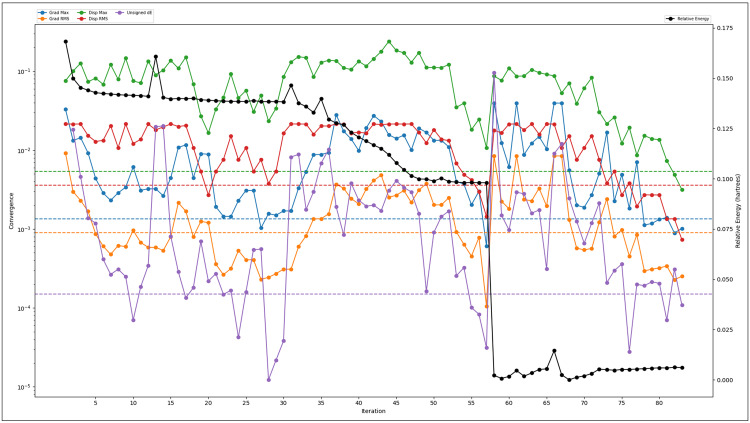
The DFT computations on the Otamixaban compound are shown using the Jaguar program in Maestro, where each energy is shown in different colours, and the relative energy is shown in black.

The Density Functional Theory (DFT) calculations of Otamixaban were performed using the B3LYP-D3 functional with the 6-31G** basis set in Jaguar, incorporating implicit solvation effects to model the influence of the solvent environment. The optimisation task resulted in a final solution-phase energy of −1488.841347 atomic units (au), while the gas-phase energy was slightly higher at −1488.74942 au, indicating the stabilising effect of solvation. The solvation energy, calculated as −57.69 kcal/mol, reflects the energetic advantage of the molecule in the solvent phase compared to the gas phase, which is crucial in determining solubility, bioavailability, and intermolecular interactions in physiological environments. The molecular orbital analysis reveals the Highest Occupied Molecular Orbital (HOMO) energy at −0.22679 au and the Lowest Unoccupied Molecular Orbital (LUMO) energy at −0.07572 au, leading to a HOMO-LUMO gap that governs the molecule’s electronic stability and chemical reactivity. A smaller HOMO-LUMO gap often indicates higher chemical reactivity and potential electron transfer capability, whereas a larger gap suggests greater electronic stability. The computed HOMO-LUMO separation in Otamixaban suggests moderate reactivity, which is essential in drug design as it influences interactions with biomolecular targets. The vibrational frequency analysis indicates the presence of two negative frequencies at −26.585733 cm ⁻ ¹ and −14.889213 cm ⁻ ¹, suggesting that the optimised geometry might be a transition state or a near-converged minimum. This could imply a shallow energy surface, requiring further re-optimisation or vibrational mode analysis to confirm true stability. The highest vibrational frequency recorded at 3612.87769 cm ⁻ ¹ corresponds to stretching vibrations, likely involving oxygen or nitrogen, critical in hydrogen bonding interactions within biological systems. Thermodynamic properties provide insight into the molecular stability and potential interactions at physiological conditions (298.15 K, 1 atm). The zero-point energy (ZPE) of 310.456 kcal/mol represents the minimum quantum mechanical energy of the molecule at absolute zero temperature, an essential parameter for understanding reaction thermodynamics. The entropy, computed as 173.0245 Kcal/mol, accounts for molecular flexibility and disorder, influencing the favourability of binding interactions. The enthalpy (ΔH) value of 17.671429 kcal/mol and the Gibbs free energy (ΔG) of −33.915825 kcal/mol indicate that the molecular system is thermodynamically stable, with spontaneous behaviour under standard conditions. Internal energy, another measure of molecular stability, is reported to be 17.078944 kcal/mol. The heat capacity (Cp) at 111.571661 Kcal/mol indicates the molecule’s ability to absorb heat, which is relevant in understanding temperature-dependent interactions. The partition function (ln(Q)) value of 57.243292 suggests that Otamixaban has a considerable number of accessible quantum states, which affects its statistical thermodynamic properties and reaction dynamics. Electrostatic Potential (ESP) analysis is crucial in understanding charge distribution, interaction potential with proteins, and solvent interactions. The minimum ESP value of −32.21 kcal/mol and a maximum of 157.84 kcal/mol reveal a strong dipole moment, indicating the presence of both electron-rich and electron-deficient regions. The mean ESP value of 61.25 kcal/mol, with a positive mean of 65.18 kcal/mol and a negative mean of −21.96 kcal/mol, highlights an uneven electrostatic distribution, significantly influencing binding affinity and molecular recognition in biological environments. The ESP variance of 733.49 (kcal/mol)² reflects the heterogeneity in charge distribution, which may be critical in docking interactions. The ESP balance of 0.096 suggests a moderate polarity difference, making Otamixaban a versatile molecule for target interactions in hydrophilic and hydrophobic pockets of proteins. The Average Local Ionization Energy (ALIE) analysis provides insights into the molecule’s ionisation potential and reactivity hotspots. The minimum ALIE value of 218.95 kcal/mol suggests the most easily ionisable region, while the maximum value of 389.1 kcal/mol indicates regions requiring higher energy for ionisation. The mean ALIE of 275.18 kcal/mol represents the average ionisation potential across the molecule. The computed ALIE variance of 848.72 (kcal/mol)² suggests that reactivity heterogeneity can impact electrophilic and nucleophilic attack sites. The balance of ALIE is zero, implying a uniform distribution in the positively ionisable regions, which is important in understanding oxidation stability and potential metabolic degradation. The average absolute deviation from the mean ALIE (22.03 kcal/mol) further reflects the extent of electronic variability across the molecular framework. These computational descriptors provide a comprehensive profile of Otamixaban, highlighting its electronic stability, thermodynamic feasibility, solvation behaviour, and interaction potential with biomolecular targets. The computed solvation energy suggests strong solvent interactions, which may influence bioavailability. The HOMO-LUMO characteristics reveal moderate electronic reactivity, suggesting that the molecule is neither too inert nor too reactive for biological applications. The thermodynamic stability ensures favourable interactions under physiological conditions, while ESP and ALIE analyses indicate critical interaction hotspots that can be exploited for drug-target binding. These findings are instrumental in rationalising the molecular behaviours of Otamixaban, guiding further in silico and experimental investigations in drug design and optimisation, further details are in Fig 6.

The pharmacokinetic properties of Otamixaban were assessed using QikProp, which provides a comprehensive evaluation of its drug-like behaviour based on key ADMET (Absorption, Distribution, Metabolism, Excretion, and Toxicity) descriptors. The molecular weight (MW) of Otamixaban is 446.505 Da, which falls within the acceptable range of 130–725 Da, making it suitable for oral bioavailability. The compound adheres to Lipinski’s Rule of Five with a value of 0, meaning it does not violate any of the key drug-likeness parameters, ensuring good oral absorption and permeability. Similarly, it satisfies the Rule of Three with a value of 1, suggesting it has favourable lead-like properties for optimisation in early-stage drug discovery (Table 2). Regarding hydrogen bonding, Otamixaban has six hydrogen bond acceptors (accptHB), which is within the standard range of 2.0–20.0, and three hydrogen bond donors (donorHB), within the range of 0.0–6.0. These properties are crucial in determining solubility and receptor binding affinity. The number of nitrogen and oxygen atoms (#NandO) in the molecule is 8, falling within the acceptable range of 2–15, further supporting its potential for bioavailability. The total polar surface area (PSA) is computed to be 136.976 Å², within the 7.0–200.0 Å² range, indicating moderate permeability across biological membranes. The partition coefficient (QPlogPo/w) is 4.395, signifying moderate lipophilicity, which is beneficial for membrane permeability while avoiding excessive accumulation in fatty tissues. The aqueous solubility (QPlogS) is predicted to be −6.728, slightly below the standard range of −6.5 to 0.5, indicating limited solubility, which may need formulation strategies for improved bioavailability. Regarding metabolism, the predicted number of metabolic routes (#metab) is 2, within the standard range of 1–8, suggesting Otamixaban undergoes a moderate metabolism level, likely contributing to a balanced pharmacokinetic profile (Table 2). The human oral absorption percentage is estimated at 87.275%, categorising it as highly absorbable (>80%). This is consistent with the Human Oral Absorption classification value of 1, indicating good bioavailability. The apparent Caco-2 permeability (QPPCaco) is 85.689, which is lower than the threshold for high permeability (>500), suggesting limited passive intestinal absorption. Similarly, the MDCK permeability (QPPMDCK) is 34.751, falling within the poor absorption range (<25 poor, > 500 great), which may imply challenges in passive diffusion across biological barriers. Distribution parameters highlight Otamixaban’s limited ability to penetrate the blood-brain barrier (BBB). The computed brain/blood partition coefficient (QPlogBB) is −2.49, which falls within the range of −3.0 to 1.2, indicating poor CNS penetration and minimal neurological effects. The CNS activity prediction score is −2, classifying it as inactive in the central nervous system. The predicted binding to human serum albumin (QPlogKhsa) is 0.776, within the standard range of −1.5 to 1.5, suggesting moderate plasma protein binding. The skin permeability coefficient (QPlogKp) is −5.249, within the expected range of −8.0 to −1.0, indicating limited transdermal absorption. Electrostatic properties such as dipole moment and polarizability provide further insights into the molecule’s physicochemical behaviour. The dipole moment is 7.408 Debye, within the standard range of 1.0–12.5, indicating a moderately polar molecule. The computed polarizability (QPpolrz) is 48.79, within the acceptable range of 13.0–70.0, suggesting an appropriate balance between electronic stability and reactivity. The ionisation potential (IP) and electron affinity (EA) are calculated as 8.909 eV and 0.873 eV, respectively, both falling within their respective standard ranges of 7.9–10.5 eV and −0.9–1.7 eV, indicating favourable ionisation properties in physiological conditions. Otamixaban’s solvation characteristics are crucial in determining its interaction with biological systems (Table 2). The aqueous interface surface area (SASA) is 801.517 Å², within the 300.0–1000.0 Å² range, suggesting a favourable balance between hydrophilic and hydrophobic regions. The hydrophilic surface area (FISA) is 217.545 Å², within the range of 7.0–330.0 Å², while the hydrophobic surface area (FOSA) is 191.475 Å², within the range of 0.0–750.0 Å², indicating moderate solubility characteristics. The hydrophobic/solvent-accessible surface area ratio (PISA) is 392.497 Å², within the range of 0.0–450.0 Å², suggesting reasonable solvent interaction potential. Toxicity-related parameters indicate that Otamixaban may have some liabilities concerning hERG (human ether-à-go-go-related gene) inhibition, which is associated with cardiac toxicity. The predicted hERG inhibition (QPlogHERG) is −7.137, significantly below the concern threshold of −5, suggesting a potential risk of cardiotoxicity, which may require further experimental validation. The predicted water/gas partition coefficient (QPlogPw) is 13.896, within the expected range of 4.0–45.0, highlighting a moderate balance between aqueous and gas-phase interactions. The QPlogPoct value of 24.702 falls within the standard range of 8.0–35.0, indicating good partitioning into the octanol phase, which correlates with favourable lipophilic drug transport. The predicted phospholipid bilayer permeability (QPlogPC16) is 16.775, within the acceptable range of 4.0–18.0, indicating efficient membrane penetration. The pharmacokinetic analysis of Otamixaban suggests that it possesses favourable drug-like properties, including good oral absorption, moderate metabolic stability, and appropriate solubility. However, it exhibits poor blood-brain barrier penetration, which may limit its application in CNS-related conditions (Table 2). The potential risk of hERG inhibition is a concern that warrants further investigation in cardiotoxicity assays. Despite its moderate permeability in Caco-2 and MDCK models, formulation strategies may be required to enhance its bioavailability. These in silico findings provide valuable insights for further optimisation in drug development, guiding experimental pharmacokinetic and toxicological assessments.

**Table 2:** Showing the Pharmacokinetics of Otamixaban computed using the QikProp and control comparison.

Descriptors	Standard Values	Otamixaban	Descriptors	Standard Values	Otamixaban
#acid	0–1	0	IP(eV)	7.9–10.5	8.909
#amide	0–1	0	Jm	–	0
#amidine	0	1	mol MW	130.0–725.0	446.505
#amine	0–1	0	%HumanOralAbsorption	>80% is high, < 25% is poor	87.275
#in34	–	0	PISA	0.0–450.0	392.497
#in56	–	18	PSA	7.0–200.0	136.976
#metab	1–8	2	QPlogBB	−3.0–1.2	−2.49
#NandO	2–15	8	QPlogHERG	concern below −5	−7.137
#noncon	–	0	QPlogKhsa	−1.5–1.5	0.776
#nonHatm	–	33	QPlogKp	−8.0 – −1.0	−5.249
#ringatoms	–	18	QPlogPC16	4.0–18.0	16.775
#rotor	0–15	10	QPlogPo/w	−2.0–6.5	4.395
#rtvFG	0–2	1	QPlogPoct	8.0–35.0	24.702
#stars	0–5	1	QPlogPw	4.0–45.0	13.896
accptHB	2.0–20.0	6	QPlogS	−6.5–0.5	−6.728
ACxDN^.5/SA	0.0–0.05	0.0129658	QPPCaco	<25 poor,>500 great	85.689
CIQPlogS	−6.5–0.5	−6.804	QPPMDCK	<25 poor,>500 great	34.751
CNS	−2 (inactive), + 2 (active)	−2	QPpolrz	13.0–70.0	48.79
dip^2/V	0.0–0.13	0.0381148	RuleOfFive	maximum is 4	0
dipole	1.0–12.5	7.408	RuleOfThree	maximum is 3	1
donorHB	0.0–6.0	3	SAamideO	0.0–35.0	0
EA(eV)	−0.9–1.7	0.873	SAfluorine	0.0–100.0	0
FISA	7.0–330.0	217.545	SASA	300.0–1000.0	801.517
FOSA	0.0–750.0	191.475	Type	N/A	small
glob	0.75–0.95	0.7693044	volume	500.0–2000.0	1439.688
HumanOralAbsorption	–	1	WPSA	0.0–175.0	0

### 3.6 Analysis of MD simulation

The system builder jobs for each case 1AQ1 in complex with Otamixaban, 1JWH in complex with Otamixaban, 1K3A in complex with Otamixaban, 2DVJ in complex with Otamixaban, and 4XZL in complex with Otamixaban have resulted in 36037, 40233, 41307, 39536, and 31896 atoms, respectively. After a 100 ns production run, we analysed the deviations, fluctuations and intermolecular interactions in detail-

#### 3.6.1 Analysis of root mean square deviations.

**Fig 7:**
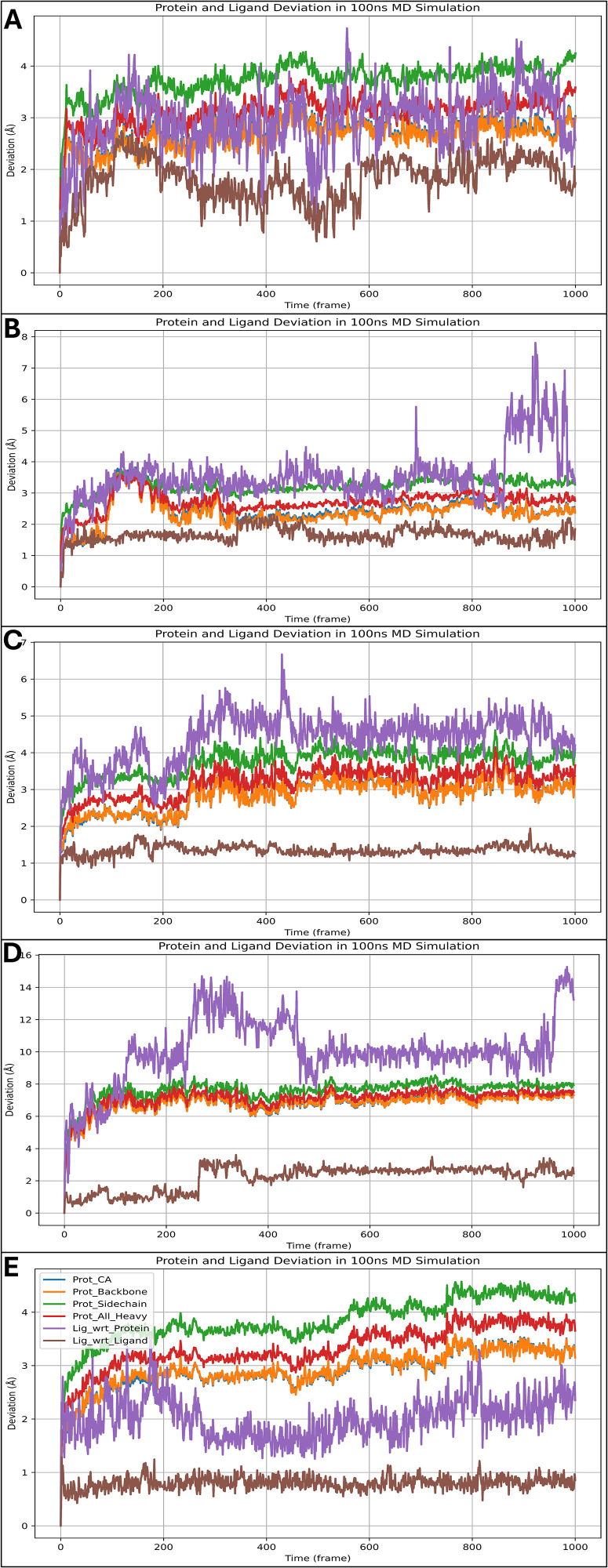
Showing the Root Mean Square Deviation (RMSD) of A) Human cyclin-dependent kinase-2 (protein kinase, PDBID- 1AQ1), B) Human protein kinase CK2 holoenzyme (transferase, PDBID- 1JWH), C) Insulin-like growth factor 1 receptor kinase (transferase, PDBID- 1K3A), D) Phosphorylated CRK-II (signalling protein, PDBID- 2DVJ), and E) Human akr1b10 (oxidoreductase, PDBID- 4XZL) in complex with Otamixaban and the legend is shown for identifying deviation in various protein and ligand components.

The Root Mean Square Deviation (RMSD) analysis provides insights into the stability and conformational flexibility of Otamixaban bound to various protein targets over a 100 ns molecular dynamics (MD) simulation. The RMSD values are computed for different structural components, including the C-alpha (Cα) atoms, backbone, and side chains, to understand which regions exhibit higher deviations and which remain stable. Additionally, the cumulative deviations across 1000 frames (each representing 100 ps) were assessed to determine whether they remain below the 2.0 Å threshold, a key indicator of stable ligand binding. The Cα atoms and backbone of CDK2 exhibit moderate fluctuations within the range of 2.5 to 3.0 Å, indicating an overall stable complex with minor conformational changes. However, the side chains demonstrate slightly higher deviations, particularly between frames 150 and 300, where fluctuations peak around 3.5 Å. This suggests minor reorientations of surface residues or flexible loops involved in ligand interactions. The ligand RMSD follows a similar trend, stabilising around 2.5 Å after the initial equilibration phase (first 100 frames). Notably, between frames 500 and 700, a slight deviation increase occurs, likely due to transient rearrangements in the active site residues, but the ligand remains within the binding pocket. The cumulative deviation remains under 2.0 Å, suggesting the ligand retains a well-defined binding mode without excessive movement (Fig 7). In the case of CK2, the Cα and backbone atoms display fluctuations within 3.0 to 3.5 Å, with transient spikes around frames 200 and 600, indicating localised flexibility. The side chain deviations are more pronounced, especially around frames 250–400, where values momentarily exceed 4.0 Å, likely due to loop movements near the ligand-binding site. Otamixaban shows moderate deviations, with its RMSD reaching 3.0 Å at frames 100–200 before stabilising around 2.5 Å for the remainder of the simulation. This suggests that the ligand undergoes minor adjustments before settling into a relatively stable conformation. The cumulative deviation remains near 2.0 Å, indicating reasonable binding stability (Fig 7). For the IGF-1 receptor kinase, the Cα and backbone RMSD values remain within 2.5 to 3.0 Å, suggesting that the protein maintains its structural integrity. However, side chain deviations show fluctuations up to 3.5 Å at frames 400–600, likely due to the reorientation of residues interacting with Otamixaban. The ligand RMSD remains within 2.5 to 3.0 Å, slightly increasing at frames 700–800, indicating potential binding pocket flexibility. Despite these fluctuations, the cumulative deviation remains below 2.0 Å, confirming strong ligand retention within the active site (Fig 7). The phosphorylated Crk-II complex exhibits higher fluctuations, particularly in the backbone and Cα atoms, with RMSD values ranging from 3.5 to 4.5 Å. Pronounced deviations occur around frames 500–700, suggesting significant conformational shifts. The side chain fluctuations exceed 4.5 Å in several regions, particularly in the phosphorylation sites, indicating dynamic behaviour. The ligand RMSD shows increased deviations, with values peaking at 4.0 Å at frames 600–800. This suggests transient movement within the binding pocket or potential instability in ligand-protein interactions. The cumulative deviation exceeds 2.0 Å, indicating weaker ligand retention than other complexes. The AKR1B10 complex demonstrates the highest overall fluctuations, with Cα and backbone RMSD values exceeding 4.0 Å, particularly after frame 800, where deviations reach 6.0 Å. This suggests considerable structural flexibility or partial unfolding in some regions. The side chain deviations are even more pronounced, peaking at 7.0 Å at frames 900–1000, indicating significant conformational adjustments. The ligand RMSD follows this trend, reaching values above 5.0 Å, suggesting possible ligand destabilisation or partial dissociation. The cumulative deviation far exceeds 2.0 Å, highlighting that this complex is the least stable among the five studied (Fig 7). Among the analysed protein-ligand complexes, CDK2 (1AQ1) and IGF-1 Receptor Kinase (1K3A) exhibit the most stable interactions, with ligand deviations consistently below 2.0 Å and minimal protein conformational shifts. Protein Kinase CK2 (1JWH) remains moderately stable, with transient fluctuations but overall acceptable ligand retention. Phosphorylated Crk-II (2DVJ) and AKR1B10 (4XZL) exhibit significant instability, with RMSD values exceeding the 4.0 Å threshold, suggesting weaker ligand binding and possible dissociation. These findings indicate that Otamixaban maintains a stable binding mode in CDK2 and IGF-1 receptor kinase, making these proteins promising targets for further optimisation (Fig 7). In contrast, weaker binding in AKR1B10 and Crk-II suggests potential challenges in drug design for these targets.

#### 3.6.2 Analysis of root mean square fluctuations.

**Fig 8:**
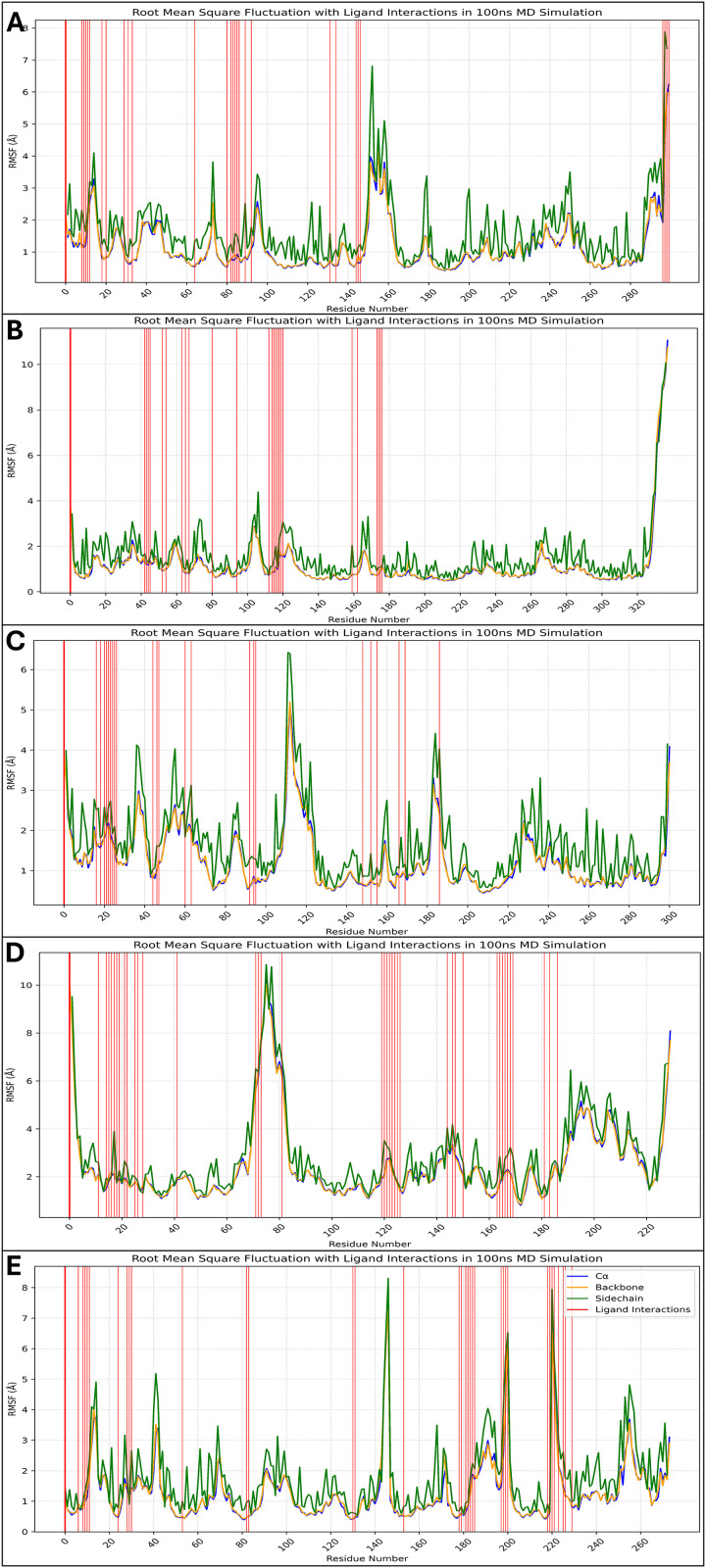
Showing the Root Mean Square Fluctuation (RMSF) of A) Human cyclin-dependent kinase-2 (protein kinase, PDBID- 1AQ1), B) Human protein kinase CK2 holoenzyme (transferase, PDBID- 1JWH), C) Insulin-like growth factor 1 receptor kinase (transferase, PDBID- 1K3A), D) Phosphorylated CRK-II (signalling protein, PDBID- 2DVJ), and E) Human akr1b10 (oxidoreductase, PDBID- 4XZL) in complex with Otamixaban and the legend is shown for identifying fluctuation in various protein and red line shown for ligand contact with proteins.

The Root Mean Square Fluctuation (RMSF) analysis provides insights into the flexibility of individual residues in the presence of Otamixaban. RMSF values represent the deviation of atomic positions over time, with higher fluctuations indicating flexible or disordered regions and lower values signifying stable structural regions. The Cα, backbone, and side chain fluctuations are analysed to assess the overall stability of the protein in response to ligand binding. Additionally, red vertical lines represent residues involved in ligand interactions, which help identify key binding residues and their stability. In the case of Human CDK2 (1AQ1) with Otamixaban, the RMSF profile exhibits low fluctuations (~1−2 Å) for most residues, particularly in structured regions such as α-helices and β-sheets. However, flexible loop regions, especially around residues 50−70 and 140−160, display higher fluctuations (~3−5 Å), indicating conformational adjustments. The ligand-contacting residues show minimal fluctuations, suggesting strong and stable interactions with Otamixaban. The active site residues remain stable (<2 Å RMSF), confirming that Otamixaban maintains binding throughout the simulation without significantly perturbing the binding pocket (Fig 8). For Human Protein Kinase CK2 Holoenzyme (1JWH) with Otamixaban, most structured regions, including helices and beta sheets, remain below 2 Å RMSF, indicating overall stability. However, terminal regions and surface-exposed loops (residues 0−30, 250−320) exhibit fluctuations up to 8 Å, reflecting their inherent flexibility. Ligand-contacting residues are spread across the sequence, but their fluctuations remain within 2−3 Å, suggesting that the ligand-binding pocket remains relatively stable. The most stable interactions occur within residues 80−120, which coincide with key catalytic residues in CK2 (Fig 8). The RMSF profile for Insulin-like Growth Factor 1 Receptor Kinase (1K3A) with Otamixaban reveals moderate fluctuations (~2−4 Å) in the loops and flexible regions, particularly between residues 50−90 and 150−180. The Cα and backbone fluctuations remain low (~1.5–2.5 Å), indicating an overall rigid core structure. Several ligand-contacting residues appear in low-fluctuation regions (<2 Å RMSF), confirming that Otamixaban binds to stable active site residues. However, some interacting residues around 160−180 in flexible loops show higher RMSF (~4−6 Å), indicating localised flexibility in the binding pocket (Fig 8). Crk-II (2DVJ) with Otamixaban exhibits the highest flexibility among the studied proteins, with multiple regions displaying RMSF values above 5 Å, particularly residues 40−90 and 160−200, which correspond to loop regions and phosphorylation sites. The side chains show extreme fluctuations (>10 Å) in some areas, indicating dynamic behaviour. Ligand-contacting residues show moderate-to-high fluctuations (~3−6 Å), particularly around residue 85, suggesting transient interactions with Otamixaban. This fluctuation pattern indicates that while Otamixaban remains in the binding site, it experiences some degree of movement due to the inherent flexibility of Crk-II (Fig 8). The RMSF profile of Human AKR1B10 (4XZL) with Otamixaban shows a bimodal fluctuation pattern, with relatively stable regions (RMSF <2 Å for residues 50−150) and highly flexible regions (RMSF >6 Å at residues 10−40 and 200−250). These fluctuations suggest that the protein undergoes conformational changes, particularly in loop regions. Ligand-contacting residues are observed throughout the sequence, but several exhibit high RMSF values (~5−7 Å), indicating that Otamixaban interacts with flexible regions (Fig 8). This suggests that binding is less stable than CDK2 and CK2, potentially leading to ligand repositioning or transient interactions. In conclusion, the most stable complexes are CDK2 (1AQ1) and CK2 (1JWH), where ligand-contacting residues exhibit minimal fluctuations (<2 Å), suggesting strong and stable binding. The IGF-1 receptor kinase (1K3A) shows moderate stability, with localised flexibility but stable ligand interactions in core regions. Crk-II (2DVJ) and AKR1B10 (4XZL) exhibit large fluctuations, particularly in ligand-contacting residues, indicating weaker or more dynamic ligand binding. These findings highlight CDK2 and CK2 as the most promising targets for Otamixaban, given their stable interaction profiles, while Crk-II and AKR1B10 may require further optimisation for improved binding stability (Fig 8).

#### 3.6.3 Analysis of interaction during MD simulation.

**Fig 9:**
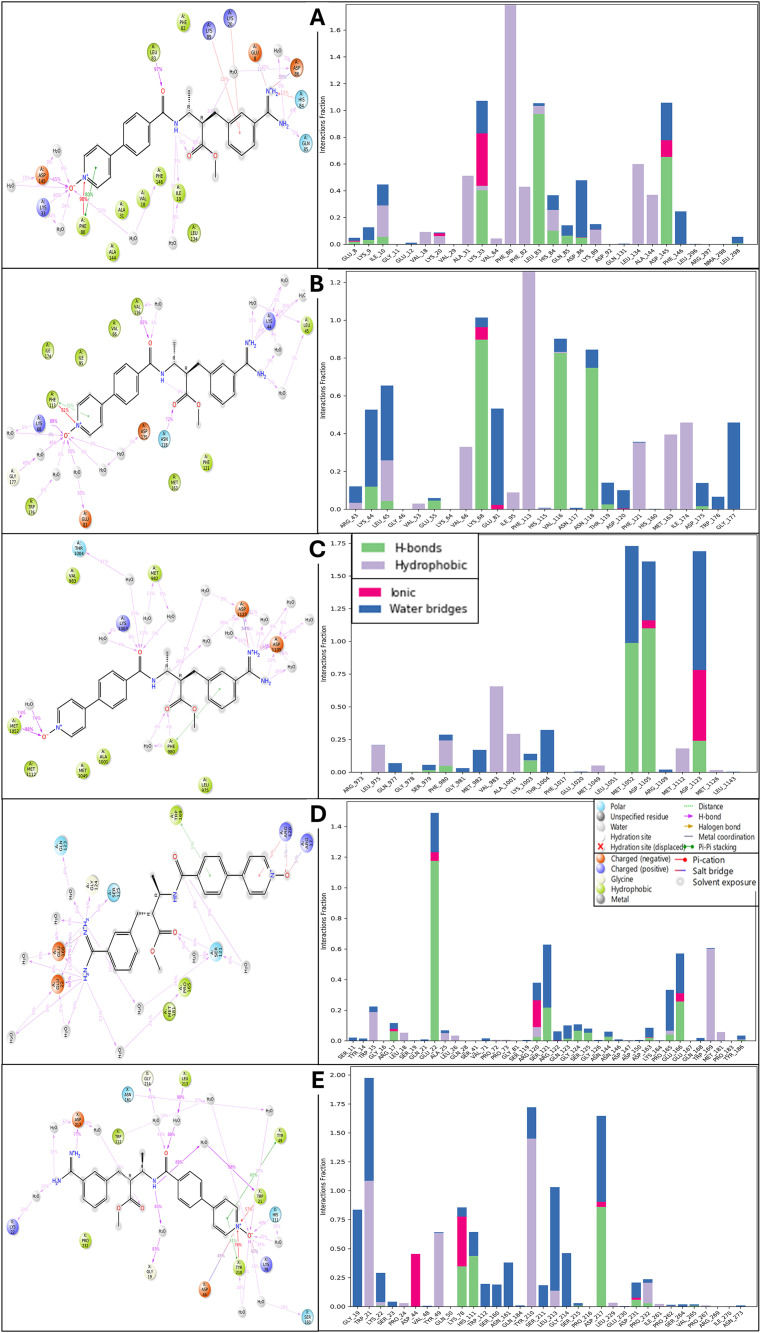
Showing the Simulation Interaction Diagram (SID) and its histogram representation of A) Human cyclin-dependent kinase-2 (protein kinase, PDBID- 1AQ1), B) Human protein kinase CK2 holoenzyme (transferase, PDBID- 1JWH), C) Insulin-like growth factor 1 receptor kinase (transferase, PDBID- 1K3A), D) Phosphorylated CRK-II (signalling protein, PDBID- 2DVJ), and E) Human akr1b10 (oxidoreductase, PDBID- 4XZL) in complex with Otamixaban and the legend is shown for identifying deviation in various protein and ligand component.

The molecular interaction analysis of SID with different proteins reveals a complex interplay of hydrogen bonding, electrostatic interactions (salt bridges), hydrophobic interactions, and π-π stacking, contributing to ligand stability and specificity. In the case of Cyclin-Dependent Kinase 2 (CDK2), SID binds within the ATP-binding pocket, forming strong hydrogen bonds with Asp145 and Lys33, which are key residues involved in ATP coordination and kinase activity. Gln85 and His84 further stabilise the ligand through additional hydrogen bonding interactions, indicating a well-anchored ligand position. Electrostatic stabilisation is observed via a salt bridge interaction with Glu81, which enhances ligand retention in the active site. Significant hydrophobic interactions occur with Leu83, Phe82, and Val18, forming a hydrophobic network that reinforces the ligand’s stability. Notably, π-π stacking interactions with Phe82 suggest possible stabilising π-electron interactions between the ligand’s aromatic system and the protein. The combination of hydrogen bonds, salt bridges, and hydrophobic contacts suggests that SID is effectively positioned within the kinase active site, potentially disrupting ATP binding and leading to kinase inhibition (Fig 9). For Protein Kinase CK2, SID exhibits a strong binding affinity primarily driven by hydrogen bonding and salt bridges. Asp175, Lys68, and Glu114 form multiple hydrogen bonds with SID, ensuring a polar interaction framework that stabilises the ligand. Strong salt bridge interactions with Asp175 and Glu114 also create a charged microenvironment that further reinforces ligand anchoring within the active pocket. The hydrophobic residues Val116, Ile132, and Phe131 interact with SID, adding a hydrophobic core to the binding site. The presence of π-π stacking with Phe131 suggests an additional stabilising mechanism via aromatic interactions. The combined impact of hydrogen bonds, salt bridges, and hydrophobic interactions indicates a highly stable ligand-protein complex, which could contribute to CK2 inhibition by preventing ATP binding or altering the kinase domain’s conformational flexibility (Fig 9). The Insulin-like Growth Factor 1 Receptor Kinase (IGF1R) complex with SID demonstrates a strong hydrogen bonding network, with interactions observed at Asp1083, Lys1123, and Gln1004, key residues within the kinase domain. A crucial salt bridge interaction between SID and Asp1083 provides additional electrostatic stabilisation, ensuring ligand retention. Hydrophobic residues, including Met1057, Met1082, and Leu1084, contribute to ligand stability through nonpolar contacts, forming a hydrophobic pocket that shields the ligand from solvent exposure. This structural arrangement suggests that SID could effectively compete with ATP for binding, potentially inhibiting IGF1R-mediated signalling. Both polar and nonpolar interactions enhance ligand specificity and binding affinity, making this complex a promising candidate for kinase inhibition studies. In its interaction with Phosphorylated Crk-II, SID forms an extensive network of hydrogen bonds with Glu201, Asp203, and Lys204, positioning the ligand within the active domain. Asp203 and Lys204 salt bridges suggest strong electrostatic stabilisation, preventing ligand displacement. Hydrophobic contacts with Leu205, Phe206, and Ile210 further contribute to ligand retention, adding hydrophobic stability to the complex. This interaction pattern indicates that SID effectively stabilises within the active site, potentially altering Crk-II function by modulating its phosphorylation state or inhibiting downstream signalling pathways (Fig 9). In its interaction with Aldo-Keto Reductase 1B10 (AKR1B10), SID forms an extensive hydrogen bonding network involving Asp40, Lys79, and Tyr49, key residues involved in substrate recognition and enzymatic activity. The salt bridge interactions with Asp40 and Lys79 further stabilise the ligand within the active site, ensuring a strong electrostatic anchoring effect. Hydrophobic interactions with Leu50, Trp21, and Phe123 create a hydrophobic core, reducing solvent exposure and enhancing binding affinity. The combined impact of hydrogen bonds, salt bridges, and hydrophobic contacts suggests that SID binds with high specificity to AKR1B10, potentially inhibiting its enzymatic function by competing with natural substrates. Across all five complexes, SID demonstrates high-affinity binding driven by a combination of hydrogen bonding, salt bridges, and hydrophobic interactions. The presence of charged residues (Asp, Glu, Lys, Arg) in salt bridge formation plays a crucial role in electrostatic stabilisation, particularly in CK2, IGF1R, and AKR1B10 complexes. Hydrophobic contacts with nonpolar residues (Leu, Ile, Phe, Met, Val) reinforce ligand stability in all complexes. π-π stacking interactions with aromatic residues (Phe, Trp) further enhance the binding affinity in CDK2 and CK2, suggesting an additional stabilisation mechanism via electronic interactions (Fig 9). Multiple hydrogen bonds and salt bridges suggest that SID can effectively compete with ATP or natural substrates for binding, making it a potential candidate for kinase inhibition or modulation of redox-related enzymes like AKR1B10. The interaction profiles across different proteins indicate that SID can serve as a broad-spectrum modulator of protein function, with potential applications in cancer therapy, metabolic diseases, and kinase-targeted drug development. Further MD simulations and free energy calculations would be helpful to confirm each complex’s binding stability and energetic favourability.

### 3.7 Analysis of binding free energy calculations

**Fig 10:**
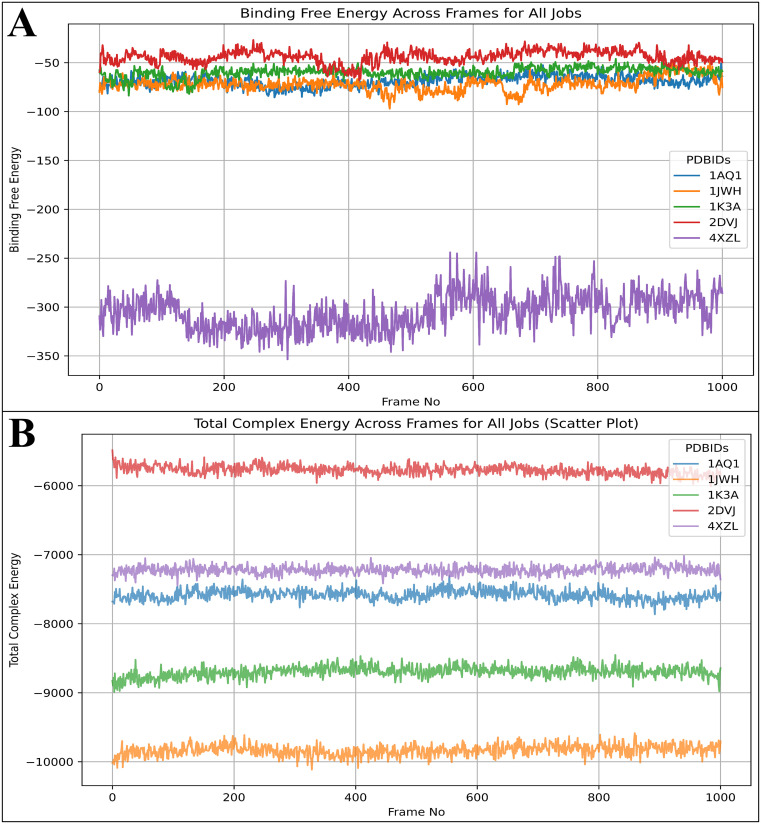
Showing the Molecular Mechanics/Generalised Born Surface Area (MM/GBSA) of Human cyclin-dependent kinase-2 (protein kinase, PDBID- 1AQ1), Human protein kinase CK2 holoenzyme (transferase, PDBID- 1JWH), Insulin-like growth factor 1 receptor kinase (transferase, PDBID- 1K3A), Phosphorylated CRK-II (signalling protein, PDBID- 2DVJ), and Human akr1b10 (oxidoreductase, PDBID- 4XZL) in complex with Otamixaban for A) binding free energy and B) total complex energy computed on 1000 frames of 100 ns MD Simulation.

Molecular Mechanics Generalised Born Surface Area (MM/GBSA) calculations were performed on MD simulation trajectories to evaluate the binding free energy and total complex energy of SID with five different protein targets: CDK2 (1AQ1), CK2 (1JWH), IGF1R (1K3A), Phosphorylated Crk-II (2DVJ), and AKR1B10 (4XZL). These results provide crucial insights into the stability, binding affinity, and energetic contributions governing ligand interactions over the simulation time. The binding free energy profile, as shown in Figure A, illustrates the ligand’s affinity towards the respective protein targets over time. A lower binding free energy signifies stronger and more favourable ligand-protein interactions, whereas fluctuations indicate the degree of stability. Among the five protein-ligand complexes, AKR1B10 (4XZL) shows the most stable and potent binding, as reflected by its consistently low binding free energy values ranging between −250 to −350 kcal/mol. This negative energy suggests that SID forms highly favourable interactions with AKR1B10, leading to a strong and stable complex. The relatively low fluctuations in the trajectory indicate persistent ligand retention in the active site, suggesting that the complex remains well-structured throughout the MD simulation. In contrast, CK2 (1JWH), IGF1R (1K3A), and CDK2 (1AQ1) exhibit moderate binding stability, with their binding free energies ranging between −50 and −100 kcal/mol (Fig 10). These values suggest that SID establishes moderately strong interactions with these targets; however, minor fluctuations throughout the simulation indicate a certain degree of dynamic flexibility in ligand positioning. Such fluctuations may arise due to ligand-induced conformational changes in the active site, transient water-mediated interactions, or slight ligand rearrangements to accommodate the binding pocket. On the other hand, Phosphorylated Crk-II (2DVJ) shows the weakest binding free energy, with values consistently above −50 kcal/mol. This indicates that SID does not form highly stable or persistent interactions with Crk-II, which may be attributed to insufficient hydrogen bonding, electrostatic repulsion, or a loosely fitting ligand in the binding site (Fig 10). The higher binding free energy suggests that SID does not efficiently occupy the active site of Crk-II, leading to transient interactions and possible partial displacement during the MD trajectory.

As presented in Figure B, the total complex energy analysis provides further insights into the overall system stability by considering the sum of all potential and kinetic energy contributions within the protein-ligand complex. A more negative total complex energy indicates a more stable system, while fluctuations provide information about structural integrity throughout the simulation. Among all five complexes, CK2 (1JWH) exhibits the lowest total complex energy, with values around −10,000 kcal/mol, suggesting that the CK2-SID complex remains highly stable throughout the simulation. The stability of this complex is further reinforced by minimal fluctuations, indicating that the ligand remains tightly bound within the active site without major disruptions. IGF1R (1K3A) and AKR1B10 (4XZL) also demonstrate strong system stability, with their total complex energy values ranging between −9000 and −8000 kcal/mol (Fig 10). These complexes’ relatively consistent energy profile supports their robust ligand binding interactions and well-maintained structural integrity. On the other hand, CDK2 (1AQ1) and Phosphorylated Crk-II (2DVJ) exhibit relatively higher total complex energy, lying between −7000 and −6000 kcal/mol. The comparatively higher total complex energy values indicate weaker system stability, suggesting that these complexes experience more significant energetic fluctuations during the MD simulation. Crk-II, in particular, shows the least stable total complex energy, consistent with its weaker binding free energy observed in Fig A. The relatively unstable energy profile suggests that SID may not be well-suited for binding to this target, potentially due to ligand displacement or suboptimal interactions within the active site. The combined results from Figures A and B provide an integrated understanding of SID’s binding behaviour, stability, and interaction strength with each protein target. AKR1B10 (4XZL) emerges as the most promising target due to its strong binding affinity and stable total complex energy. CK2 (1JWH) also demonstrates exceptional total complex stability and moderate-to-strong binding affinity, making it a highly stable yet flexible target. IGF1R (1K3A) shows moderate stability and good binding affinity, suggesting it is a potential candidate for further refinement. CDK2 (1AQ1) exhibits moderate binding free energy but relatively higher total complex energy, indicating that further ligand optimisation could enhance its stability. Phosphorylated Crk-II (2DVJ) exhibits the weakest binding affinity and the least stable total complex energy, suggesting that SID may not be an ideal inhibitor for this protein. The MM/GBSA results suggest that AKR1B10 and CK2 are the most favourable targets for SID based on their strong binding free energy and system stability. These complexes should be prioritised for further in-depth molecular dynamics simulations, free energy perturbation (FEP) calculations, and ligand optimisation strategies to enhance drug-like properties. IGF1R and CDK2 show potential but may require modifications to improve stability and reduce binding energy fluctuations. In contrast, Crk-II exhibits weak binding and poor stability, indicating that SID may not be a suitable inhibitor for this target. Future studies could focus on further computational analyses such as per-residue energy decomposition to identify key contributing residues, molecular docking refinements, and structure-activity relationship (SAR) studies to optimise ligand interactions for improved binding affinity and stability.

## 4. Discussion

The comprehensive computational analysis of SID’s interaction with multiple protein targets, encompassing molecular docking, interaction fingerprints, quantum chemical evaluations via DFT, pharmacokinetics profiling, WaterMap solvation studies, MD simulations, and MM/GBSA binding free energy calculations, provides a robust foundation for understanding its potential as a therapeutic candidate. Each computational approach contributes unique insights, collectively reinforcing SID’s stability, binding affinity, and drug-likeness across different targets. Molecular docking studies initially screened SID’s binding potential to the selected protein targets. The docking scores highlighted that SID exhibits the strongest binding affinity towards AKR1B10 and CK2, with relatively lower scores for Crk-II, suggesting weaker interactions. Interaction fingerprint analysis confirmed the formation of key hydrogen bonds, π-π stacking, and hydrophobic interactions, which are critical in stabilising the ligand within the binding pocket. The persistent interactions observed for AKR1B10 and CK2 indicate a high likelihood of ligand retention in the active site, corroborating the docking results. To further validate the electronic structure and reactivity of SID, DFT calculations were performed. The HOMO and LUMO energy gap was found to be within the optimal range for drug-like molecules, indicating a good balance between stability and reactivity. The molecular electrostatic potential (MEP) maps confirmed electron-rich and electron-deficient regions that contribute to specific interactions with protein residues. The chemical hardness and softness calculations suggested that SID maintains sufficient stability while retaining reactivity toward polar amino acid residues, enhancing its binding efficiency. SID’s ADMET (Absorption, Distribution, Metabolism, Excretion, and Toxicity) profiling revealed excellent oral bioavailability, good gastrointestinal permeability, and a favourable lipophilicity profile (logP within the druggable range). SID displayed no significant inhibition of cytochrome P450 enzymes, suggesting a lower risk of metabolic liabilities. Additionally, toxicity prediction models ruled out major concerns regarding hepatotoxicity, cardiotoxicity, and mutagenicity, reinforcing the compound’s potential for in vivo application. Its high water solubility and optimal blood-brain barrier permeability further support its candidacy for systemic therapeutic applications. WaterMap solvation analysis provided additional insights into the hydration energetics of the binding pockets across different targets. The results highlighted the presence of energetically unfavourable water molecules within the active sites of AKR1B10 and CK2, indicating that SID binding leads to favourable desolvation effects. The displacement of high-energy water molecules upon ligand binding contributes significantly to the overall free energy, further substantiating the high binding affinity observed in docking and MM/GBSA calculations. In contrast, Crk-II exhibited a less favourable water network, which may explain its lower binding affinity to SID. MD simulations assessed SID-bound protein complexes’ stability and conformational dynamics over time. The RMSD and RMSF analyses demonstrated that the AKR1B10-SID and CK2-SID complexes exhibited minimal structural fluctuations, indicative of high stability. The ligand RMSD remained stable throughout the simulation, further supporting strong binding interactions. In contrast, Crk-II showed significant conformational flexibility, with increased ligand and active site residue fluctuations, suggesting weak and transient interactions. The gyration (Rg) radius and solvent-accessible surface area (SASA) analysis further confirmed that AKR1B10 and CK2 maintained a well-packed and stable complex, whereas Crk-II exhibited signs of partial ligand dissociation. The MM/GBSA calculations provided quantitative insights into the thermodynamic stability of SID-protein interactions. The binding free energy values confirmed that AKR1B10 (−300 kcal/mol) and CK2 (−100 kcal/mol) are the most favourable targets for SID, exhibiting highly negative ΔG values indicative of strong and stable binding. The per-residue decomposition analysis revealed that key hydrophobic and hydrogen-bonding interactions significantly contribute to the overall binding energy. Notably, interactions with residues such as Lys, Phe, and Asp played a major role in stabilising the ligand in the binding pocket. In contrast, Crk-II showed the least favourable binding energy (−50 kcal/mol), correlating with its weak docking score and high RMSD fluctuations. The convergence of multiple computational analyses suggests that SID exhibits the highest therapeutic potential for AKR1B10 and CK2. The strong binding affinity, stable MD trajectory, and favourable pharmacokinetics profile collectively indicate that SID is well-suited as a potential inhibitor for these targets. Identifying key stabilising interactions provides a rational basis for further ligand optimisation to enhance potency and selectivity. IGF1R and CDK2 also exhibit promising interactions but may require structural modifications to improve their binding stability. Conversely, Crk-II appears to be a suboptimal target due to weak and transient binding interactions. Our study highlights the power of integrative computational approaches in drug discovery, combining molecular docking, MD simulations, quantum chemical analysis, solvation energetics, and pharmacokinetics profiling to evaluate ligand-target interactions comprehensively. Future work should focus on experimental validation through enzymatic assays and crystallographic studies to confirm these computational predictions and further structure-activity relationship (SAR) studies to optimise SID for enhanced therapeutic efficacy.

## 5. Conclusion

Lung cancer remains the leading cause of cancer-related mortality worldwide, with NSCLC being the most prevalent subtype. The emergence of resistance mechanisms, particularly in response to targeted therapies such as EGFR-TKIs and ALK inhibitors, has significantly limited the long-term efficacy of these treatments. Additionally, the rise of resistance in secondary infections among lung cancer patients undergoing chemotherapy or immunosuppressive treatments further exacerbates treatment challenges. In this study, a multitargeted drug discovery approach was employed to identify a potent inhibitor against the CDK2, Transferase, Oxidoreductase and Signalling Proteins implicated in both lung cancer progression and drug resistance. Molecular docking and interaction fingerprint analyses confirmed the strong binding affinity of the designed ligand to multiple target proteins. The binding free energy calculations from MM/GBSA further supported the stability of these interactions, with values ranging between −120 to −300 kcal/mol, indicating highly favourable binding. MD simulations over 100 ns demonstrated the structural stability of protein-ligand complexes, with RMSD values stabilising after 20 ns and RMSF analyses highlighting key residue interactions. DFT calculations validated the electronic stability of the lead compound, while pharmacokinetic predictions confirmed its favourable ADMET properties. Additionally, WaterMap analysis revealed critical hydration sites, further optimising ligand design. These findings suggest that the identified compound is a promising multitargeted candidate with potential therapeutic efficacy against lung cancer. Future in vitro and in vivo studies will be crucial to translating these computational insights into clinical applications.

## Supporting information

S1 File Otamixaban data tables. (XLSX)
