# Correction: Evaluating the pentapharmacological potency of otamixaban against lung cancer CDK2, transferase, oxidoreductase and signalling proteins

**DOI:** 10.1371/journal.pone.0344592

**Published:** 2026-03-09

**Authors:** Hanadi M. Baeissa, Tahani Bakhsh, Afnan Mohammed Shakoori, Ghadir Sindi, Randa Mohammed Zaki, Mohammad Azhar Kamal, Hadeel A. Alsufyani, Alaa Hamed Habib, Arwa Ishaq A. Khayyat, Misbahuddin Rafeeq, Deeba Shamim Jairajpuri

The Funding statement for this article is incorrect. The correct Funding statement is as follows: This study is supported by Prince Sattam bin Abdulaziz University, project number PSAU/2024/R/1445
